# Mass spectrometry-based characterisation of the cardiac microtissue metabolome and lipidome

**DOI:** 10.1007/s11306-025-02252-0

**Published:** 2025-04-21

**Authors:** Tara J. Bowen, Andrew R. Hall, Andrew D. Southam, Ossama Edbali, Ralf J. M. Weber, Amanda Wilson, Amy Pointon, Mark R. Viant

**Affiliations:** 1 School of Biosciences, University of Birmingham, Edgbaston, Birmingham, B15 2TT UK; 2 Safety Sciences, Clinical Pharmacology and Safety Sciences, BioPharmaceuticals R&D, AstraZeneca, Cambridge, UK; 3 Phenome Centre Birmingham, University of Birmingham, Edgbaston, Birmingham, B15 2TT UK; 4 Integrated Bioanalysis, Clinical Pharmacology and Safety Sciences, BioPharmaceuticals R&D, AstraZeneca, Cambridge, UK; 5 Present Address: Medicines Discovery Catapult, Alderley Park, Cheshire, SK10 4TG UK

**Keywords:** Metabolomics, Metabolome annotation, Cardiac microtissues, UHPLC-MS

## Abstract

**Introduction:**

The use of large, non-sample specific metabolite reference libraries often results in high proportions of false positive annotations in untargeted metabolomics.

**Objective:**

This study aimed to measure and curate a library of polar metabolites and lipids present in cardiac microtissues.

**Results:**

Untargeted ultra-high performance liquid chromatography-coupled mass spectrometry measurements of cardiac microtissue intracellular extracts were annotated by comparison against four spectral databases and a retention time library. The annotations were combined to create a library of 313 polar metabolites and 1004 lipids.

**Conclusions:**

The curated library will facilitate higher confidence metabolite annotation in mass spectrometry-based untargeted metabolomics of cardiac microtissues.

**Supplementary Information:**

The online version contains supplementary material available at 10.1007/s11306-025-02252-0.

## Introduction

The metabolite annotation of spectral features is integral to the application of untargeted metabolomics for toxicology and safety assessment. For example, it has been proposed that predictive metabolic biomarkers, if to be used individually, need to be identified to Metabolomics Standards Initiative (MSI) level 1 (Malinowska & Viant, 2019). Also, mechanistic interpretation of metabolomics data, e.g., through pathway enrichment analysis relies on good coverage of correctly annotated metabolites (Wieder et al., 2021). Yet despite its importance, metabolite annotation remains a major bottleneck in untargeted metabolomics (Nash & Dunn, 2019).

The cause of the annotation challenge in mass spectrometry (MS)-based metabolomics is multifactorial but includes difficulties in deconvoluting the highly complex data, i.e., due to measurement of multiple signals per metabolite, and distinguishing between isomers. Moreover, the public metabolite databases, e.g., Human Metabolome Database (HMDB) (Wishart et al., 2021), Kyoto Encyclopaedia of Genes and Genomes (KEGG) (Kanehisa, 2000), and LIPID MAPS^®^ Structure Database (LMSD) (Sud et al., 2007), often used as the search space for annotation purposes, contain many compounds that are either not present or not detectable in the sample type that has been analysed. This can give rise to an increased number of putative annotations associated with a single feature and a much greater probability of false positive annotations (Nash & Dunn, 2019). A proposed solution to overcome, in part, the challenge of annotation in untargeted metabolomics is the replacement of large, diverse metabolite databases, e.g., HMDB (Wishart et al., 2021), with smaller, accurately defined lists of metabolites known to be present and detectable within the specific sample type, for use as the search space. In principle, this would reduce the proportion of false positive annotations attained (Nash & Dunn, 2019). To this end, Deep Metabolome Annotation (DMA) of test systems, i.e., experimentally and accurately define which metabolites are present and detectable within a specific sample type, has been recommended (Viant et al., 2017). Consistent with this recommendation, a comprehensive approach combining solid phase extraction, untargeted ultra-high performance liquid chromatography coupled mass spectrometry (UHPLC-MS(/MS), gas chromatography-MS (GC-MS), nuclear magnetic resonance (NMR) spectroscopy and nano-electrospray direct infusion MS^n^ was applied for DMA of *Daphnia magna* (Jones, 2017). However, the approaches typically implemented for DMA, e.g., as applied by Jones (2017) are time-consuming and expensive, limiting their routine application. Other, less comprehensive, attempts at annotating metabolomes extend to the human adult urinary metabolome (Roux et al., 2012), zebrafish embryo metabolome (Xu et al., 2021), and bovine liver and heart lipidomes (Hackbusch et al., 2019) where researchers have achieved good metabolome coverage through use of complementary LC-MS(/MS) assays (Roux et al., 2012; Xu et al., 2021) and intelligent data-dependent acquisition (DDA) strategies for MS^n^ acquisition (Hackbusch et al., 2019).

The aim of this study was to curate a library of polar metabolites and lipids present in cardiac microtissues and which are detectable by electrospray ionisation-MS, to facilitate higher confidence annotations of untargeted metabolomics datasets derived from cardiac microtissues. To achieve this aim, concentrated intracellular polar metabolite and lipid extracts, each from the equivalent of ca. 375 cardiac microtissues, were analysed by hydrophilic interaction (HILIC) and C_30_-based reverse phase (RP-C_30_) UHPLC-MS, respectively, using an intelligent DDA workflow (AcquireX, Thermo Scientific) to maximise the coverage of fragmentation data. The second objective was to employ an in-house retention time (RT) library and MS/MS spectral databases of toxicologically-relevant metabolites (MTox700 + list of toxicity biomarkers) (Sostare et al., 2022), mzCloud™ (Thermo Scientific) and two in silico lipid spectral databases, LipidSearch™ (Thermo Scientific) and LipidBlast (Kind et al., 2013), to annotate the detectable polar metabolome and lipidome of cardiac microtissues. Finally, the curated lists were explored with respect to coverage of metabolic pathways and lipid classes, and toxicological relevance.

## Methods

### Sample generation and analysis

Fourteen day mature cardiac microtissues were harvested 2- and 72-hours after a media change into samples of 154-pooled microtissues, as described previously (Bowen et al., 2021, Supplementary Information). Intracellular polar metabolites and lipids were extracted using 4:1 (v/v) methanol/water and 2:1 (v/v) methanol/chloroform, respectively. Extracts were pooled, aliquoted, and concentrated by drying and reconstitution in 1.5:1.5:1.0 (v/v/v) acetonitrile/methanol/water for polar metabolomics by HILIC UHPLC-MS or 3:1 (v/v) isopropanol/water for lipidomics by RP-C_30_ UHPLC-MS (Bowen et al., 2021). Sample analysis was performed using an Orbitrap ID-X Tribrid mass spectrometer (Thermo Scientific) coupled to a Vanquish Horizon UHPLC (Thermo Scientific) using previously reported methods with some modifications (Jankevics et al., 2021; Southam et al., 2020). An AcquireX Deep Scan (Thermo Scientific) workflow was implemented for intelligent DDA of MS^n^ fragmentation data. Further details are provided in Supplementary Information (SI).

### Data processing and annotation

Data processing and annotation was performed using:

1. XCMS (v3.14.0, Smith et al., 2006) to generate a peak-intensity matrix for features (*m/z*-RT pairs) vs. samples for each dataset (Table S2). Data matrices were subsequently filtered to remove features of non-biological origin and non-reproducible features: only features detected in full scan injections of microtissue extract from at least two of three AcquireX cycles, with intensity greater than 10x the blank median, were retained. The Bioconductor/R package msPurity (v1.18.0) (Lawson et al., 2017) was implemented to process (precursor ion purity > 0.5; SNR of fragment ions > 3), and average (across all samples, keeping only fragment ions in ≥ 50% contributing spectra) MS^2^ data, generating a database of MS^2^ spectra per assay. Annotations were subsequently assigned by comparison of the generated databases to an in-house database of MS^2^ spectra using the msPurity spectral matching function (dot product cosine score > 0.6).
2. Compound Discoverer™ (v3.3, Thermo Scientific). This included peak detection, deconvolution, background subtraction, and MS/MS-based spectral matching to mzCloud™, LipidBlast (Kind et al., 2013), and libraries contributed by Prof. Bamba (Kyushu University) (Table S3). The reported compound tables were filtered to retain only compounds detected in at least two of three AcquireX cycles, with at least MS/MS data, a RT > 1-minute, and with spectral match score (HighChem HighRes) against an mzCloud or mzVault record > 0.6.
3. LipidSearch™ (v5.0, Thermo Scientific). Data acquired by RP-C_30_ assays were processed and annotated using the LipidSearch in silico database (Table S4). The reported annotations were filtered, retaining only those annotated in at least two of three AcquireX cycles, and assigned a grade of A, B or C (Table S5).

RT matching was also performed. Annotations were assigned where *m/z* and RTs of features matched (± 5 ppm and ± 40-seconds tolerance, respectively) those of reference standards (MTox700+, Sostare et al., 2022).

Automatically assigned annotations were manually inspected and curated (see SI for the lipid annotation acceptance criteria). Where metabolites were annotated based on MS/MS spectral match to public database only, the annotation confidence may be defined as MSI Level 2 (referred to as a high confidence annotation; Sumner et al., 2007). MSI Level 1 confidence was defined where at least *m/z* and RT matched those of an authentic chemical standard that was analysed using the same analytical parameters (referred to as an identification).

Pathway mapping against the KEGG Homo sapiens KEGG pathway library was performed using MetaboAnalyst (v5.0, https://www.metaboanalyst.ca/MetaboAnalyst/*)* Pathway Analysis function.

## Results and discussion

### MS^n^ coverage

UHPLC-MS^n^ measurements of intracellular metabolite and lipid extracts from cardiac microtissues were first assessed with respect to the coverage of MS^n^ data. Alignment of MS/MS fragmentation spectra processed and filtered by msPurity to the filtered XCMS-derived feature list revealed fragmentation data were available for 631, 619, 2942 and 2562 features from the HILIC positive, HILIC negative, RP-C_30_ positive and RP-C_30_ negative datasets, respectively (Table S6). This is equivalent to approximately 44–62% coverage of the experimental datasets. Meanwhile, MS^2^ data were acquired for 80–90% of filtered compounds reported by Compound Discoverer™ (Table S6). This may be considered very high coverage, consistent with previous reports of AcquireX coverage capabilities (Thermo Scientific), and further demonstrating the benefits of applying intelligent DDA.

### Polar metabolites

Combining annotations derived from spectral matching against mzCloud™ and an in-house spectral database, and RT matching against an in-house RT library of metabolic biomarkers for toxicology (MTox700+, Sostare et al., 2022), a list of 313 compounds present in polar intracellular extracts of cardiac microtissues was curated (Table S7). A total of 126 of the reported compounds were identified (MSI Level 1, Sumner et al., 2007). The remaining 187 compounds were high confidence annotations only (MSI Level 2, Sumner et al., 2007), thus some caution should be taken in forming biological interpretations from the curated list alone. Examination of the curated list indicates the presence of metabolites associated with key functions in cardiac biochemistry. For example, the inclusion of the branched chain amino acids (BCAA), leucine, isoleucine and valine, an alternative group of substrates to fatty acids and glucose for cardiac energy metabolism and ATP synthesis. Impaired BCAA metabolism has been associated with multiple cardiovascular diseases, including myocardial hypertrophy, cardiomyopathies and heart failure (Xiong et al., 2022). As a further example, the inclusion of creatine, which is involved in cardiac contraction via its phosphorylation to phosphocreatine, providing a mechanism for effective transportation and rapid synthesis of ATP. The creatine/phosphocreatine system is of particular importance at times of increased energy demand or oxygen deficiency (Balestrino, 2021).

Notably, the list contains 124 lipids, including 11 acylcarnitines, 1 ceramide, 39 lysophospholipids, 64 phospholipids, and 9 sphingomyelins (Table S7). The presence of these compounds in polar extracts is consistent with findings of Southam et al. (2020), where lipids were also measured in acetonitrile/methanol/water-based monophasic extracts of mammalian tissue by HILIC UHPLC-MS. This may be explained by the absence of a non-polar phase, as occurs in a biphasic extraction, into which most lipids would otherwise partition.

**Fig. 1:**
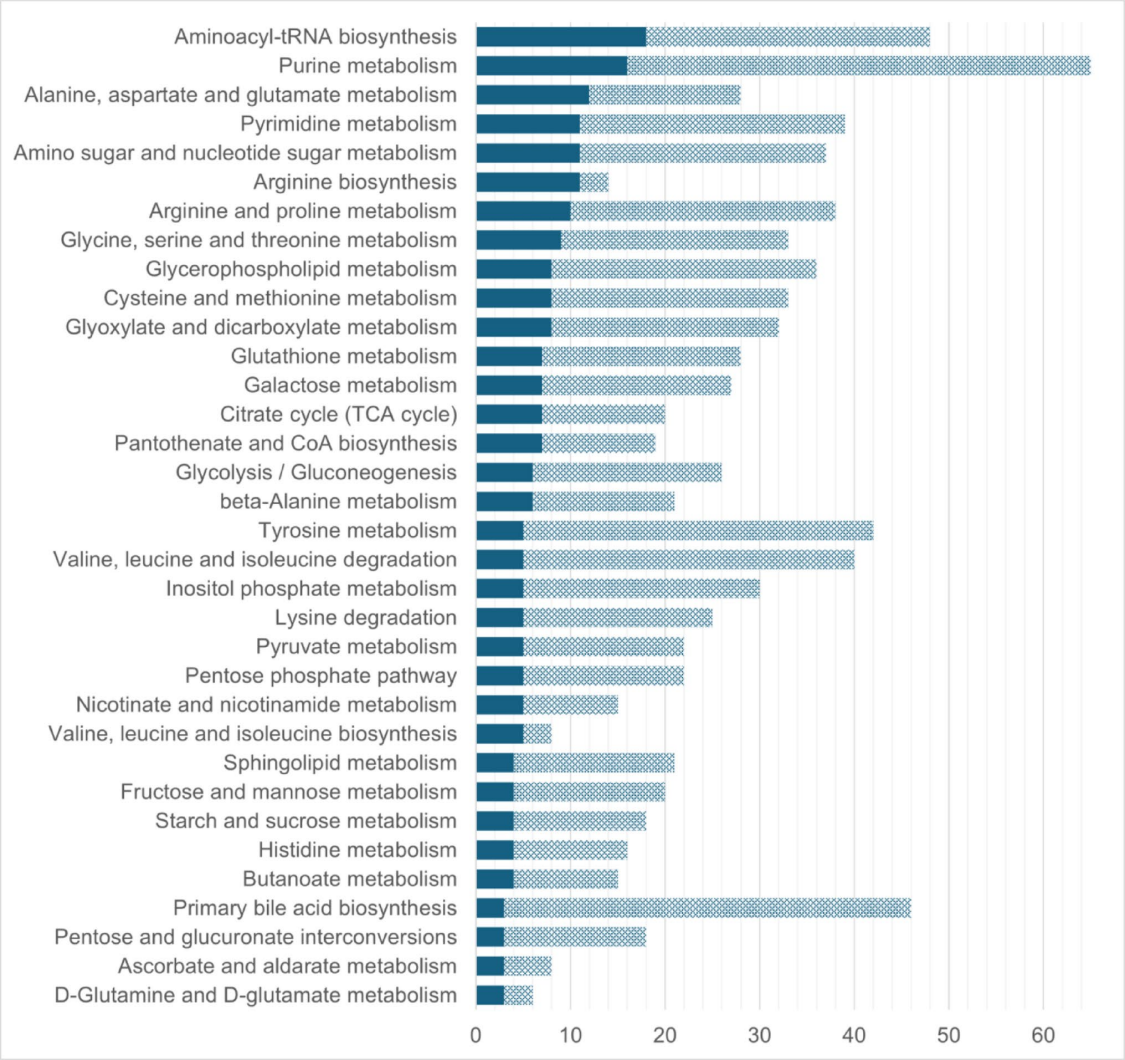
Metabolic pathways represented by the curated list of polar metabolites. Plot shows the names of KEGG biochemical pathways and the number of metabolites within the curated list of polar metabolites detected in intracellular extracts of cardiac microtissues which mapped to each pathway (solid bars). The total number of metabolites within each pathway is represented by the sum of the solid and hashed bars. Only pathways with representation by at least three metabolites within the curated list of polar metabolites are shown. Full list of represented pathways can be found in Table S8

The list of polar compounds was interrogated further by mapping against the KEGG database of biochemical pathways. A total of 204 of the metabolites were mapped, revealing representation of 57 (human) KEGG pathways (Fig. 1, Table S8). The most represented pathway, based on the number of mapped compounds, is the aminoacyl-tRNA biosynthesis pathway. Aminoacyl-tRNAs are responsible for delivering amino acids to the ribosome for synthesis of proteins by translation (Rubio Gomez & Ibba, 2020). The detection and annotation of 18 amino acids within polar intracellular extracts of cardiac microtissues is responsible for this being the most represented pathway (Fig. 1, Tables S7-S8). Other well represented pathways include purine metabolism and pyrimidine metabolism (Fig. 1, Table S8). The generation of nucleotides is essential for cell proliferation, while altered nucleotide metabolism is associated with cardiotoxicity induced by anthracyclines (Asnani et al., 2020). Additionally, the representation of central energy metabolism pathways, including the citrate cycle, glycolysis/gluconeogenesis and pentose phosphate pathway, is pertinent to the application of untargeted metabolomic analysis to cardiac microtissues with the aim of discovering effects of structural cardiotoxins since many have previously been reported to disrupt cardiac energy metabolism (Force & Kolaja, 2011).

The curated list of polar compounds was also compared against the MTox700 + list of metabolic biomarkers for toxicology (Sostare et al., 2022), revealing 54.6% of the detected and annotated compounds have previously been associated with toxicity (Table S7). The presence of compounds considered to be predictive of toxicological endpoints supports the use of cardiac microtissues for metabolomics-based discovery of biomarkers predictive of structural cardiotoxicity.

### Lipids

A list of 1004 unique lipid groups, i.e., reporting fatty acyl chains as total number of carbons and double bonds, equivalent to ‘abbreviated names’ in LMSD (Sud et al., 2007), present in intracellular lipid extracts of cardiac microtissues was curated from annotations generated by spectral matching against LipidBlast in silico MS/MS database (Kind et al., 2013), MS^2^ and MS^3^-based annotation using LipidSearch™, and msPurity-based spectral matching against an in-house MS/MS database (Table S9, Fig. S3). Twenty-one of the reported lipids were identified where the accurate mass, retention time and MS/MS data matched that of an analytical standard. The remaining 983 lipids were annotated only; some caution should be taken when forming biological interpretations from these results.

**Fig. 2:**
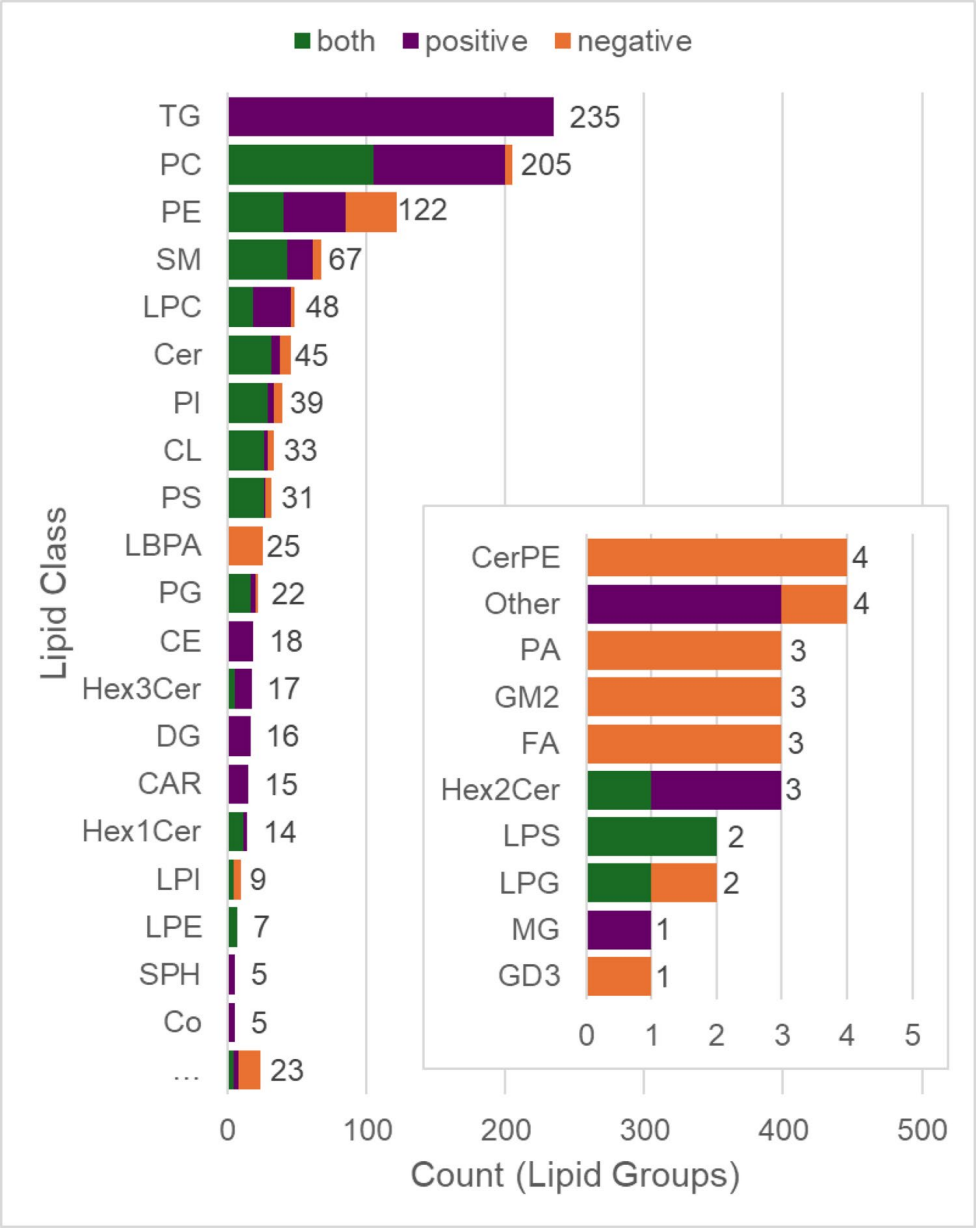
Abundance of lipids according to class. The number of lipids (lipid groups) per lipid class (LipidSearch™ nomenclature) within the curated list of annotated compounds detected in lipid extracts of cardiac microtissues, and the ionisation mode by which they were measured (purple: positive only; orange: negative only; green: both). Common name definitions of lipid class acronyms are as follows, *PC* phosphatidylcholine, *TG* triglyceride; *PE* phosphatidylethanolamine; *PA* phosphatidic acid; *PG* phosphatidylglycerol; *SM* sphingomyelin; *CL* cardiolipin; *PS* phosphatidylserine; *LPC* lyso-PC; *DG* diacylglycerol; *Cer* ceramide; *PI* phosphatidylinositol; *LPE* lyso-PE; *LBPA* monoacylglycerophosphomonoacylglycerols; *Hex1Cer* hexosylceramide; *Hex2Cer* dihexosylceramide; *CE* cholesterol ester; *Hex3Cer* trihexosyl ceramide; *CAR* fatty acylcarnitine; *LPG* lyso-PG; *LPI* lyso-PI; *SPH* sphingoid bases; *LPS* lyso-PS; *Co* coenzyme; *CerPE* PE-ceramide; *GM2* ganglioside GM2; *MG*: monoacylglycerol; GD3; *FA* fatty acyl; *LPA* lyso-PA; *GD3* ganglioside GD3

The most abundant lipid class according to the number of annotations was triglycerides, followed by phosphatidylcholines (Fig. 2). These findings are consistent with phosphatidylcholines being the most abundant phospholipid of mammalian cells and subcellular organelles (Van der Veen et al., 2017). Meanwhile, triglycerides serve as the major energy supply for the heart. Alterations in the utilisation of triglycerides, including accumulation due to reduced fatty acyl oxidation, are associated with impaired cardiac function (Goldberg et al., 2012). The accumulation of other lipid classes, including diglycerides and ceramides, which are involved in signalling, and acylcarnitines, which shuttle fatty acyls into the mitochondria for β-oxidation, has also been linked to cardiotoxicity (Goldberg et al., 2012). Therefore, the detection and annotation of these lipids within intracellular extracts of cardiac microtissues is pertinent to discovering metabolic biomarkers of cardiotoxicity.

## Conclusion

This study provides a valuable resource for metabolomics experiments utilising cardiac microtissues as the biological test system, e.g., in toxicology. The curated lists of 313 polar metabolites and 1004 lipid groups, which encompass cardiac energy substrates (e.g., BCAAs), intermediates (e.g., creatine, acylcarnitines) and stores (e.g., triglycerides), and central building blocks of macromolecules (e.g., amino acids, nucleotides) and cell membranes (e.g., phospholipids), are expected to facilitate higher confidence annotation by yielding fewer false positives when used in place of larger, non-sample specific public metabolite databases, e.g., HMDB. Further efforts to achieve definitive identifications of all metabolites and lipids reported by the analysis of analytical standards would provide greater confidence in this resource and in any resultant biological interpretations. More broadly, the approaches described here, when applied to generate metabolite libraries specific to other toxicological test systems, have the potential to enhance the value of untargeted and hybrid metabolomics across toxicology and safety assessment.

## Electronic supplementary material

Below is the link to the electronic supplementary material.

Supplementary Material 1

Supplementary Material 2

Supplementary Material 3

## Data Availability

Data is provided within the manuscript or supplementary information files. Raw and transformed UHPLC-MS(/MS) data and full metadata from this study will be made available in MetaboLights (accession code: MTBLS11412).
