# Supplementary material for: Mass spectrometry-based characterisation of the cardiac microtissue metabolome and lipidome
Source: Metabolomics. 2025 Apr 21;21(3):54. doi: 10.1007/s11306-025-02252-0 (PMC12011886; doi:10.1007/s11306-025-02252-0)
Supplement: Supplementary file 1 — Supplementary Material 1 [file 11306_2025_2252_MOESM1_ESM.pdf]

Created by https://lipidomicstandards.org, version v2.4.0

Contents of Report

Separation Workflow 1
Overall study design . . . 1
Lipid extraction . . . 1
Analytical platform . . . 1
Quality control . . . 1
Method qualification and validation . . . 2
Reporting . . . 2

Sample Descriptions 2
Cardiac microtissues / Human / Cells . . . 2

Lipid Class Descriptions 3
1) CAR[M+H]+ / Lipid identification . . . 3
1) CAR[M+H]+ / Lipid quantification . . . 3

Separation Workflow

Overall study design

Title of the study	Mass spectrometry-based characterisation of the cardiac microtissue metabolome and lipidome		
Document creation date	10/28/2024	Corresponding Email	mark.viant@bham.ac.uk
Principal investigator	Tara Bowen	Is the workflow targeted or untargeted?	Untargeted
Institution	University of Birmingham	Clinical	No

Lipid extraction

Extraction method	1-phase system	Were internal standards added prior extraction?	No
pH adjustment	None	Special conditions	-
1-phase system	2:1 methanol/chloroform	Derivatization	-

Analytical platform

Ionizaton additives	Ammonium formate	MS Level	MSn
Number of separation dimensions	One dimension	Mass window for precursor ion isolation (in Da total isolation window)	1
Separation type 1	LC	Mass resolution for detected ion at MSn	High resolution
Separation mode 1 (liquid)	RP	Resolution at m/z 200 at MSn	30000
Detector	Mass spectrometer	Mass accuracy in ppm at MSn	5
MS type	Orbitrap	Recording mode of raw data at MSn	Centroid mode
MS vendor	Thermo	Was/Were additional dimension/techniques used	No
Ion source	ESI		

Quality control

Blanks	Yes	Quality control	No
Type of Blanks	Extraction blank, Solvent blank		

Method qualification and validation

Method validation	No

Reporting

Are reported raw data uploaded into repository?	Yes	Summary data	Identification data
Link to repository / ID to entry	MTBLS11412	Raw data upload	Yes
Are metadata available?	Yes	Additional comments	-

Sample Descriptions

Cardiac microtissues / Human / Cells

Storage and collection conditions	Available	Freeze-thaw cycles	1
Provided preanalytical information	Freeze-thaw cycles	Additives	None
Temperature handling original sample	4-8 °C	Were samples stored under inert gas?	No
Instant sample preparation	No	Additional preservation methods	No
Storage temperature	-80 °C	Biobank samples	No

Lipid Class Descriptions

1) CAR[M+H]+ / Lipid identification

Lipid class	CAR	Did you presume assumptions for identification?	No
MS Level for identification	MS1, MS2	Check on:	-
Identification level	Molecular species level	Limit of detection	S/N ratio > 3
Polarity mode	Positive	RT verified by standard	No
Type of positive (precursor)ion	[M+H]+	Separation of isobaric/isomeric interferece confirmed	No
Fragments for identification	Fragment name: NL[R1]; M+H-N(CH3)3; NL[R1+NMe3]	Model for separation prediction	No
Isotope correction at MS1	No	Additional dimension/techniques	-
Isotope correction at MS2	No	Lipid Identification Software	LipidSearch
MS1 verified by standard	No	Data manipulation	Smoothing, Centroiding, Background subtraction
MS2 verified by standard	No	Nomenclature for intact lipid molecule	Yes
Background check at MS1	Yes	Nomenclature for fragment ions	N/A
Background check at MS2	Yes	Further identification remarks	-

1) CAR[M+H]+ / Lipid quantification

Quantitative	No	Batch correction	No
Normalization to reference	No	Further quantification remarks	-

2) Cer[M+H]+ / Lipid identification

Lipid class	Cer	Did you presume assumptions for identification?	No
MS Level for identification	MS1, MS2	Check on:	-
Identification level	Molecular species level	Limit of detection	S/N ratio > 3
Polarity mode	Positive	RT verified by standard	No
Type of positive (precursor)ion	[M+H]+	Separation of isobaric/isomeric interferece confirmed	No
Fragments for identification		Model for separation prediction	No

Fragment name
-(H2O,18)
SPH(R1)+H-H2O
SPH(R1)+H-2H2O
SPH(R1)+H-H2O-CH2O

Isotope correction at MS1	No	Additional dimension/techniques	-
Isotope correction at MS2	No	Lipid Identification Software	LipidSearch
MS1 verified by standard	No	Data manipulation	Smoothing, Centroiding, Background subtraction
MS2 verified by standard	No	Nomenclature for intact lipid molecule	Yes
Background check at MS1	No	Nomenclature for fragment ions	N/A
Background check at MS2	No	Further identification remarks	-

2) Cer[M+H]+ / Lipid quantification

Quantitative	No	Batch correction	No
Normalization to reference	No	Further quantification remarks	-

3) CE[M+NH4]+ / Lipid identification

Lipid class	CE	Did you presume assumptions for identification?	No
MS Level for identification	MS1, MS2	Check on:	-
Identification level	Molecular species level	Limit of detection	S/N ratio > 3
Polarity mode	Positive	RT verified by standard	No
Type of positive (precursor)ion	[M+NH4]+	Separation of isobaric/isomeric interferece confirmed	No
Fragments for identification<br>**Fragment name**<br>M-FA		Model for separation prediction	No
Isotope correction at MS1	No	Additional dimension/techniques	-
Isotope correction at MS2	No	Lipid Identification Software	LipidSearch
MS1 verified by standard	No	Data manipulation	Smoothing, Centroiding, Background subtraction
MS2 verified by standard	No	Nomenclature for intact lipid molecule	Yes
Background check at MS1	Yes	Nomenclature for fragment ions	N/A
Background check at MS2	No	Further identification remarks	-

3) CE[M+NH4]+ / Lipid quantification

Quantitative	No	Batch correction	No
Normalization to reference	No	Further quantification remarks	-

4) BMP[M+NH4]+ / Lipid identification

Lipid class	BMP	Did you presume assumptions for identification?	No
MS Level for identification	MS1, MS2	Check on:	-
Identification level	Molecular species level	Limit of detection	S/N ratio > 3
Polarity mode	Positive	RT verified by standard	No
Type of positive (precursor)ion	[M+NH4]+	Separation of isobaric/isomeric interferece confirmed	No
Fragments for identification<br>**Fragment name**<br>FA1<br>FA2		Model for separation prediction	No
Isotope correction at MS1	No	Additional dimension/techniques	-
Isotope correction at MS2	No	Lipid Identification Software	LipidSearch
MS1 verified by standard	No	Data manipulation	Smoothing, Centroiding, Background subtraction
MS2 verified by standard	No	Nomenclature for intact lipid molecule	Yes
Background check at MS1	Yes	Nomenclature for fragment ions	N/A
Background check at MS2	No	Further identification remarks	-

4) BMP[M+NH4]+ / Lipid quantification

Quantitative	No	Batch correction	No
Normalization to reference	No	Further quantification remarks	-

5) DG[M+NH4]+ / Lipid identification

Lipid class	DG	Did you presume assumptions for identification?	No
MS Level for identification	MS1, MS2	Check on:	-
Identification level	Molecular species level	Limit of detection	S/N ratio > 3
Polarity mode	Positive	RT verified by standard	No
Type of positive (precursor)ion	[M+NH4]+	Separation of isobaric/isomeric interferece confirmed	No
Fragments for identification		Model for separation prediction	No
Isotope correction at MS1	No	Additional dimension/techniques	-
Isotope correction at MS2	No	Lipid Identification Software	LipidSearch
MS1 verified by standard	No	Data manipulation	Smoothing, Centroiding, Background subtraction
MS2 verified by standard	No	Nomenclature for intact lipid molecule	Yes
Background check at MS1	Yes	Nomenclature for fragment ions	N/A
Background check at MS2	No	Further identification remarks	-

Fragment name
-FA1(-H)-(H2O+NH3)
-FA2(-H)-(H2O+NH3)
-(H2O+NH3,35)

5) DG[M+NH4]+ / Lipid quantification

Quantitative	No	Batch correction	No
Normalization to reference	No	Further quantification remarks	-

6) DG[M+Na]+ / Lipid identification

Lipid class	DG	Did you presume assumptions for identification?	No
MS Level for identification	MS1, MS2	Check on:	-
Identification level	Molecular species level	Limit of detection	S/N ratio > 3
Polarity mode	Positive	RT verified by standard	No
Type of positive (precursor)ion	[M+Na]+	Separation of isobaric/isomeric interferece confirmed	No
Fragments for identification		Model for separation prediction	No

Fragment name
NL[R1]
NL[R2]

Isotope correction at MS1	No	Additional dimension/techniques	-
Isotope correction at MS2	No	Lipid Identification Software	LipidSearch
MS1 verified by standard	No	Data manipulation	Smoothing, Centroiding, Background subtraction
MS2 verified by standard	No	Nomenclature for intact lipid molecule	Yes
Background check at MS1	Yes	Nomenclature for fragment ions	N/A
Background check at MS2	No	Further identification remarks	-

6) DG[M+Na]+ / Lipid quantification

Quantitative	No	Batch correction	No
Normalization to reference	No	Further quantification remarks	-

7) Hex2Cer[M+H]+ / Lipid identification

Lipid class	Hex2Cer	Did you presume assumptions for identification?	No
MS Level for identification	MS1, MS2	Check on:	-
Identification level	Molecular species level	Limit of detection	S/N ratio > 3
Polarity mode	Positive	RT verified by standard	No
Type of positive (precursor)ion	[M+H]+	Separation of isobaric/isomeric interferece confirmed	No
Fragments for identification		Model for separation prediction	No

Fragment name
-(H2O,18)
SPH(R1)-H2O
NL[G2]
NL[G2,H2O]

Isotope correction at MS1	No	Additional dimension/techniques	-
Isotope correction at MS2	No	Lipid Identification Software	LipidSearch
MS1 verified by standard	No	Data manipulation	Smoothing, Centroiding, Background subtraction
MS2 verified by standard	No	Nomenclature for intact lipid molecule	Yes
Background check at MS1	Yes	Nomenclature for fragment ions	N/A
Background check at MS2	No	Further identification remarks	-

7) Hex2Cer[M+H]+ / Lipid quantification

Quantitative	No	Batch correction	No
Normalization to reference	No	Further quantification remarks	-

8) Hex3Cer[M+H]+ / Lipid identification

Lipid class	Hex3Cer	Did you presume assumptions for identification?	No
MS Level for identification	MS1, MS2	Check on:	-
Identification level	Molecular species level	Limit of detection	S/N ratio > 3
Polarity mode	Positive	RT verified by standard	No
Type of positive (precursor)ion	[M+H]+	Separation of isobaric/isomeric interferece confirmed	No
Fragments for identification		Model for separation prediction	No

Fragment name
-(H2O,18)
NL[G3]
NL[G3,H2O]
SPH(R1)-H2O

Isotope correction at MS1	No	Additional dimension/techniques	-
Isotope correction at MS2	No	Lipid Identification Software	LipidSearch
MS1 verified by standard	No	Data manipulation	Smoothing, Centroiding, Background subtraction
MS2 verified by standard	No	Nomenclature for intact lipid molecule	Yes
Background check at MS1	Yes	Nomenclature for fragment ions	N/A
Background check at MS2	No	Further identification remarks	-

8) Hex3Cer[M+H]+ / Lipid quantification

Quantitative	No	Batch correction	No
Normalization to reference	No	Further quantification remarks	-

9) LPC[M+H]+ / Lipid identification

Lipid class	LPC	Did you presume assumptions for identification?	No
MS Level for identification	MS1, MS2	Check on:	-
Identification level	Molecular species level	Limit of detection	S/N ratio > 3
Polarity mode	Positive	RT verified by standard	No
Type of positive (precursor)ion	[M+H]+	Separation of isobaric/isomeric interferece confirmed	No
Fragments for identification		Model for separation prediction	No

Fragment name
HG(PC,184)
(C5H13NO,104)
-HG(PC,183)
(C5H13NO,104)-(H2O)

Isotope correction at MS1	No	Additional dimension/techniques	-
Isotope correction at MS2	No	Lipid Identification Software	LipidSearch
MS1 verified by standard	No	Data manipulation	Smoothing, Centroiding, Background subtraction
MS2 verified by standard	No	Nomenclature for intact lipid molecule	Yes
Background check at MS1	Yes	Nomenclature for fragment ions	N/A
Background check at MS2	No	Further identification remarks	-

9) LPC[M+H]+ / Lipid quantification

Quantitative	No	Batch correction	No
Normalization to reference	No	Further quantification remarks	-

10) LPC O[M+H]+ / Lipid identification

Lipid class	LPC O	Did you presume assumptions for identification?	No
MS Level for identification	MS1, MS2	Check on:	-
Identification level	Molecular species level	Limit of detection	S/N ratio > 3
Polarity mode	Positive	RT verified by standard	No
Type of positive (precursor)ion	[M+H]+	Separation of isobaric/isomeric interferece confirmed	No
Fragments for identification	Fragment name: -(H2O); HG(PC,86); HG(PC,184); (C5H13NO,104)	Model for separation prediction	No
Isotope correction at MS1	No	Additional dimension/techniques	-
Isotope correction at MS2	No	Lipid Identification Software	LipidSearch
MS1 verified by standard	No	Data manipulation	Smoothing, Centroiding, Background subtraction
MS2 verified by standard	No	Nomenclature for intact lipid molecule	Yes
Background check at MS1	Yes	Nomenclature for fragment ions	N/A
Background check at MS2	No	Further identification remarks	-

10) LPC O[M+H]+ / Lipid quantification

Quantitative	No	Batch correction	No
Normalization to reference	No	Further quantification remarks	-

11) LPC P[M+H]+ / Lipid identification

Lipid class	LPC P	Did you presume assumptions for identification?	No
MS Level for identification	MS1, MS2	Check on:	-
Identification level	Molecular species level	Limit of detection	S/N ratio > 3
Polarity mode	Positive	RT verified by standard	No
Type of positive (precursor)ion	[M+H]+	Separation of isobaric/isomeric interferece confirmed	No
Fragments for identification		Model for separation prediction	No

Fragment name
HG(PC,184)
(C5H13NO,104)

Isotope correction at MS1	No	Additional dimension/techniques	-
Isotope correction at MS2	No	Lipid Identification Software	LipidSearch
MS1 verified by standard	No	Data manipulation	Smoothing, Centroiding, Background subtraction
MS2 verified by standard	No	Nomenclature for intact lipid molecule	No
Background check at MS1	Yes	Nomenclature for fragment ions	N/A
Background check at MS2	No	Further identification remarks	-

11) LPC P[M+H]+ / Lipid quantification

Quantitative	No	Batch correction	No
Normalization to reference	No	Further quantification remarks	-

12) LPE[M+H]+ / Lipid identification

Lipid class	LPE	Did you presume assumptions for identification?	No
MS Level for identification	MS1, MS2	Check on:	-
Identification level	Molecular species level	Limit of detection	S/N ratio > 3
Polarity mode	Positive	RT verified by standard	No
Type of positive (precursor)ion	[M+H]+	Separation of isobaric/isomeric interferece confirmed	No
Fragments for identification		Model for separation prediction	No

Fragment name
-HG(PE,141)
-FA1(+H)

Isotope correction at MS1	No	Additional dimension/techniques	-
Isotope correction at MS2	No	Lipid Identification Software	LipidSearch
MS1 verified by standard	No	Data manipulation	Smoothing, Centroiding, Background subtraction
MS2 verified by standard	No	Nomenclature for intact lipid molecule	No
Background check at MS1	Yes	Nomenclature for fragment ions	N/A
Background check at MS2	No	Further identification remarks	-

12) LPE[M+H]+ / Lipid quantification

Quantitative	No	Batch correction	No
Normalization to reference	No	Further quantification remarks	-

13) LPI[M+Na]+ / Lipid identification

Lipid class	LPI	Did you presume assumptions for identification?	No
MS Level for identification	MS1, MS2	Check on:	-
Identification level	Molecular species level	Limit of detection	S/N ratio > 3
Polarity mode	Positive	RT verified by standard	No
Type of positive (precursor)ion	[M+Na]+	Separation of isobaric/isomeric interferece confirmed	No
Fragments for identification		Model for separation prediction	No

Fragment name
-HG(PI,260)
FA1

Isotope correction at MS1	No	Additional dimension/techniques	-
Isotope correction at MS2	No	Lipid Identification Software	LipidSearch
MS1 verified by standard	No	Data manipulation	Smoothing, Centroiding, Background subtraction
MS2 verified by standard	No	Nomenclature for intact lipid molecule	No
Background check at MS1	Yes	Nomenclature for fragment ions	N/A
Background check at MS2	No	Further identification remarks	-

13) LPI[M+Na]+ / Lipid quantification

Quantitative	No	Batch correction	No
Normalization to reference	No	Further quantification remarks	-

14) MG[M+NH4]+ / Lipid identification

Lipid class	MG	Did you presume assumptions for identification?	No
MS Level for identification	MS1, MS2	Check on:	-
Identification level	Molecular species level	Limit of detection	S/N ratio > 3
Polarity mode	Positive	RT verified by standard	No
Type of positive (precursor)ion	[M+NH4]+	Separation of isobaric/isomeric interferece confirmed	No
Fragments for identification		Model for separation prediction	No

Fragment name
FA1
R1-OH

Isotope correction at MS1	No	Additional dimension/techniques	-
Isotope correction at MS2	No	Lipid Identification Software	LipidSearch
MS1 verified by standard	No	Data manipulation	Smoothing, Centroiding, Background subtraction
MS2 verified by standard	No	Nomenclature for intact lipid molecule	No
Background check at MS1	Yes	Nomenclature for fragment ions	N/A
Background check at MS2	No	Further identification remarks	-

14) MG[M+NH4]+ / Lipid quantification

Quantitative	No	Batch correction	No
Normalization to reference	No	Further quantification remarks	-

15) PC[M+H]+ / Lipid identification

Lipid class	PC	Did you presume assumptions for identification?	No
MS Level for identification	MS1, MS2	Check on:	-
Identification level	Molecular species level	Limit of detection	S/N ratio > 3
Polarity mode	Positive	RT verified by standard	No
Type of positive (precursor)ion	[M+H]+	Separation of isobaric/isomeric interferece confirmed	No
Fragments for identification		Model for separation prediction	No

Fragment name
HG(PC,184)
(C5H13NO,104)
(C5H13NO,104)-(H2O)
-FA2(+H)

Isotope correction at MS1	No	Additional dimension/techniques	-
Isotope correction at MS2	No	Lipid Identification Software	LipidSearch
MS1 verified by standard	No	Data manipulation	Smoothing, Centroiding, Background subtraction
MS2 verified by standard	No	Nomenclature for intact lipid molecule	No
Background check at MS1	Yes	Nomenclature for fragment ions	N/A
Background check at MS2	No	Further identification remarks	-

15) PC[M+H]+ / Lipid quantification

Quantitative	No	Batch correction	No
Normalization to reference	No	Further quantification remarks	-

16) PC O[M+H]+ / Lipid identification

Lipid class	PC O	Did you presume assumptions for identification?	No
MS Level for identification	MS1, MS2	Check on:	-
Identification level	Molecular species level	Limit of detection	S/N ratio > 3
Polarity mode	Positive	RT verified by standard	No
Type of positive (precursor)ion	[M+H]+	Separation of isobaric/isomeric interferece confirmed	No
Fragments for identification		Model for separation prediction	No

Fragment name
HG(PC,184)
104
NL[PC]

Isotope correction at MS1	No	Additional dimension/techniques	-
Isotope correction at MS2	No	Lipid Identification Software	LipidSearch
MS1 verified by standard	No	Data manipulation	Smoothing, Centroiding, Background subtraction
MS2 verified by standard	No	Nomenclature for intact lipid molecule	No
Background check at MS1	Yes	Nomenclature for fragment ions	N/A
Background check at MS2	No	Further identification remarks	-

16) PC O[M+H]+ / Lipid quantification

Quantitative	No	Batch correction	No
Normalization to reference	No	Further quantification remarks	-

17) PE[M+H]+ / Lipid identification

Lipid class	PE	Did you presume assumptions for identification?	No
MS Level for identification	MS1, MS2	Check on:	-
Identification level	Molecular species level	Limit of detection	S/N ratio > 3
Polarity mode	Positive	RT verified by standard	No
Type of positive (precursor)ion	[M+H]+	Separation of isobaric/isomeric interferece confirmed	No
Fragments for identification		Model for separation prediction	No

Fragment name
-HG(PE,141)
-FA1(+H)
MG(R1)-OH
MG(R2)-OH

Isotope correction at MS1	No	Additional dimension/techniques	-
Isotope correction at MS2	No	Lipid Identification Software	LipidSearch
MS1 verified by standard	No	Data manipulation	Smoothing, Centroiding, Background subtraction
MS2 verified by standard	No	Nomenclature for intact lipid molecule	No
Background check at MS1	Yes	Nomenclature for fragment ions	N/A
Background check at MS2	No	Further identification remarks	-

17) PE[M+H]+ / Lipid quantification

Quantitative	No	Batch correction	No
Normalization to reference	No	Further quantification remarks	-

18) PE O[M+H]+ / Lipid identification

Lipid class	PE O	Did you presume assumptions for identification?	No
MS Level for identification	MS1, MS2	Check on:	-
Identification level	Molecular species level	Limit of detection	S/N ratio > 3
Polarity mode	Positive	RT verified by standard	No
Type of positive (precursor)ion	[M+H]+	Separation of isobaric/isomeric interferece confirmed	No
Fragments for identification		Model for separation prediction	No

Fragment name
-HG(PE,141)
MG(R1)-OH

Isotope correction at MS1	No	Additional dimension/techniques	-
Isotope correction at MS2	No	Lipid Identification Software	LipidSearch
MS1 verified by standard	No	Data manipulation	Smoothing, Centroiding, Background subtraction
MS2 verified by standard	No	Nomenclature for intact lipid molecule	No
Background check at MS1	Yes	Nomenclature for fragment ions	N/A
Background check at MS2	No	Further identification remarks	-

18) PE O[M+H]+ / Lipid quantification

Quantitative	No	Batch correction	No
Normalization to reference	No	Further quantification remarks	-

19) LPE P[M+H]+ / Lipid identification

Lipid class	LPE P	Did you presume assumptions for identification?	No
MS Level for identification	MS1, MS2	Check on:	-
Identification level	Molecular species level	Limit of detection	S/N ratio > 3
Polarity mode	Positive	RT verified by standard	No
Type of positive (precursor)ion	[M+H]+	Separation of isobaric/isomeric interferece confirmed	No
Fragments for identification		Model for separation prediction	No

Fragment name
-HG(PE,141)

Isotope correction at MS1	No	Additional dimension/techniques	-
Isotope correction at MS2	No	Lipid Identification Software	LipidSearch
MS1 verified by standard	No	Data manipulation	Smoothing, Centroiding, Background subtraction
MS2 verified by standard	No	Nomenclature for intact lipid molecule	No
Background check at MS1	Yes	Nomenclature for fragment ions	N/A
Background check at MS2	No	Further identification remarks	-

19) LPE P[M+H]+ / Lipid quantification

Quantitative	No	Batch correction	No
Normalization to reference	No	Further quantification remarks	-

20) PG[M+H]+ / Lipid identification

Lipid class	PG	Did you presume assumptions for identification?	No
MS Level for identification	MS1, MS2	Check on:	-
Identification level	Molecular species level	Limit of detection	S/N ratio > 3
Polarity mode	Positive	RT verified by standard	No
Type of positive (precursor)ion	[M+H]+	Separation of isobaric/isomeric interferece confirmed	No
Fragments for identification		Model for separation prediction	No
Isotope correction at MS1	No	Additional dimension/techniques	-
Isotope correction at MS2	No	Lipid Identification Software	LipidSearch
MS1 verified by standard	No	Data manipulation	Smoothing, Centroiding, Background subtraction
MS2 verified by standard	No	Nomenclature for intact lipid molecule	No
Background check at MS1	Yes	Nomenclature for fragment ions	N/A
Background check at MS2	No	Further identification remarks	-

Fragment name
-HG(PG,172+H)
LPG(G1)-OH

20) PG[M+H]+ / Lipid quantification

Quantitative	No	Batch correction	No
Normalization to reference	No	Further quantification remarks	-

21) PI[M+H]+ / Lipid identification

Lipid class	PI	Did you presume assumptions for identification?	No
MS Level for identification	MS1, MS2	Check on:	-
Identification level	Molecular species level	Limit of detection	S/N ratio > 3
Polarity mode	Positive	RT verified by standard	No
Type of positive (precursor)ion	[M+H]+	Separation of isobaric/isomeric interferece confirmed	No
Fragments for identification		Model for separation prediction	No

Fragment name
-HG(PI,260+H)
MG[R2]-OH
MG[R1]-OH

Isotope correction at MS1	No	Additional dimension/techniques	-
Isotope correction at MS2	No	Lipid Identification Software	LipidSearch
MS1 verified by standard	No	Data manipulation	Smoothing, Centroiding, Background subtraction
MS2 verified by standard	No	Nomenclature for intact lipid molecule	No
Background check at MS1	Yes	Nomenclature for fragment ions	N/A
Background check at MS2	No	Further identification remarks	-

21) PI[M+H]+ / Lipid quantification

Quantitative	No	Batch correction	No
Normalization to reference	No	Further quantification remarks	-

22) PS[M+H]+ / Lipid identification

Lipid class	PS	Did you presume assumptions for identification?	No
MS Level for identification	MS1, MS2	Check on:	-
Identification level	Molecular species level	Limit of detection	S/N ratio > 3
Polarity mode	Positive	RT verified by standard	No
Type of positive (precursor)ion	[M+H]+	Separation of isobaric/isomeric interferece confirmed	No
Fragments for identification		Model for separation prediction	No

Fragment name
-HG(PS,185)
MG(R1)-OH
MG(R2)-OH

Isotope correction at MS1	No	Additional dimension/techniques	-
Isotope correction at MS2	No	Lipid Identification Software	LipidSearch
MS1 verified by standard	No	Data manipulation	Smoothing, Centroiding, Background subtraction
MS2 verified by standard	No	Nomenclature for intact lipid molecule	No
Background check at MS1	Yes	Nomenclature for fragment ions	N/A
Background check at MS2	No	Further identification remarks	-

22) PS[M+H]+ / Lipid quantification

Quantitative	No	Batch correction	No
Normalization to reference	No	Further quantification remarks	-

23) SM[M+H]+ / Lipid identification

Lipid class	SM	Did you presume assumptions for identification?	No
MS Level for identification	MS1, MS2	Check on:	-
Identification level	Molecular species level	Limit of detection	S/N ratio $>$ 3
Polarity mode	Positive	RT verified by standard	No
Type of positive (precursor)ion	[M+H]+	Separation of isobaric/isomeric interferece confirmed	No
Fragments for identification		Model for separation prediction	No

Fragment name
HG(PC,184)
-(H2O,18)
-(HG,201)

Isotope correction at MS1	No	Additional dimension/techniques	-
Isotope correction at MS2	No	Lipid Identification Software	LipidSearch
MS1 verified by standard	No	Data manipulation	Smoothing, Centroiding, Background subtraction
MS2 verified by standard	No	Nomenclature for intact lipid molecule	No
Background check at MS1	Yes	Nomenclature for fragment ions	N/A
Background check at MS2	No	Further identification remarks	-

23) SM[M+H]+ / Lipid quantification

Quantitative	No	Batch correction	No
Normalization to reference	No	Further quantification remarks	-

24) SPB[M+H]+ / Lipid identification

Lipid class	SPB	Did you presume assumptions for identification?	No
MS Level for identification	MS1, MS2	Check on:	-
Identification level	Molecular species level	Limit of detection	S/N ratio $>$ 3
Polarity mode	Positive	RT verified by standard	No
Type of positive (precursor)ion	[M+H]+	Separation of isobaric/isomeric interferece confirmed	No
Fragments for identification		Model for separation prediction	No

Fragment name
-(H2O,18)

Isotope correction at MS1	No	Additional dimension/techniques	-
Isotope correction at MS2	No	Lipid Identification Software	LipidSearch
MS1 verified by standard	No	Data manipulation	Smoothing, Centroiding, Background subtraction
MS2 verified by standard	No	Nomenclature for intact lipid molecule	No
Background check at MS1	Yes	Nomenclature for fragment ions	N/A
Background check at MS2	No	Further identification remarks	-

24) SPB[M+H]+ / Lipid quantification

Quantitative	No	Batch correction	No
Normalization to reference	No	Further quantification remarks	-

25) SE[M+H]+ / Lipid identification

Lipid class	SE	Did you presume assumptions for identification?	No
MS Level for identification	MS1, MS2	Check on:	-
Identification level	Molecular species level	Limit of detection	S/N ratio > 3
Polarity mode	Positive	RT verified by standard	No
Type of positive (precursor)ion	[M+H]+	Separation of isobaric/isomeric interferece confirmed	No
Fragments for identification<br>**Fragment name**<br>M-FA		Model for separation prediction	No
Isotope correction at MS1	No	Additional dimension/techniques	-
Isotope correction at MS2	No	Lipid Identification Software	LipidSearch
MS1 verified by standard	No	Data manipulation	Smoothing, Centroiding, Background subtraction
MS2 verified by standard	No	Nomenclature for intact lipid molecule	No
Background check at MS1	Yes	Nomenclature for fragment ions	N/A
Background check at MS2	No	Further identification remarks	-

25) SE[M+H]+ / Lipid quantification

Quantitative	No	Batch correction	No
Normalization to reference	No	Further quantification remarks	-

26) TG[M+NH4]+ / Lipid identification

Lipid class	TG	Did you presume assumptions for identification?	No
MS Level for identification	MS1, MS2	Check on:	-
Identification level	Molecular species level	Limit of detection	S/N ratio > 3
Polarity mode	Positive	RT verified by standard	No
Type of positive (precursor)ion	[M+NH4]+	Separation of isobaric/isomeric interferece confirmed	No
Fragments for identification		Model for separation prediction	No

Fragment name
FA1
FA2
FA3
-FA1(+HO)-(NH3)
-FA2(+HO)-(NH3)
-FA3(+HO)-(NH3)

Isotope correction at MS1	No	Additional dimension/techniques	-
Isotope correction at MS2	No	Lipid Identification Software	LipidSearch
MS1 verified by standard	No	Data manipulation	Smoothing, Centroiding, Background subtraction
MS2 verified by standard	No	Nomenclature for intact lipid molecule	No
Background check at MS1	Yes	Nomenclature for fragment ions	N/A
Background check at MS2	No	Further identification remarks	-

26) TG[M+NH4]+ / Lipid quantification

Quantitative	No	Batch correction	No
Normalization to reference	No	Further quantification remarks	-

27) WE[M+NH4]+ / Lipid identification

Lipid class	WE	Did you presume assumptions for identification?	No
MS Level for identification	MS1, MS2	Check on:	-
Identification level	Molecular species level	Limit of detection	S/N ratio > 3
Polarity mode	Positive	RT verified by standard	No
Type of positive (precursor)ion	[M+NH4]+	Separation of isobaric/isomeric interferece confirmed	No
Fragments for identification		Model for separation prediction	No

Fragment name
R1
R2

Isotope correction at MS1	No	Additional dimension/techniques	-
Isotope correction at MS2	No	Lipid Identification Software	LipidSearch
MS1 verified by standard	No	Data manipulation	Smoothing, Centroiding, Background subtraction
MS2 verified by standard	No	Nomenclature for intact lipid molecule	No
Background check at MS1	Yes	Nomenclature for fragment ions	N/A
Background check at MS2	No	Further identification remarks	-

27) WE[M+NH4]+ / Lipid quantification

Quantitative	No	Batch correction	No
Normalization to reference	No	Further quantification remarks	-

28) Cer[M+HCOO]- / Lipid identification

Lipid class	Cer	Did you presume assumptions for identification?	No
MS Level for identification	MS1, MS2	Check on:	-
Identification level	Molecular species level	Limit of detection	S/N ratio > 3
Polarity mode	Negative	RT verified by standard	No
Type of negative (precursor)ion	[M+HCOO]-	Separation of isobaric/isomeric interferece confirmed	No
Fragments for identification		Model for separation prediction	No

Fragment name
LCB(-CH3O)
FA1(+C2H3N)
FA1(+C2H3NO)

Isotope correction at MS1	No	Additional dimension/techniques	-
Isotope correction at MS2	No	Lipid Identification Software	LipidSearch
MS1 verified by standard	No	Data manipulation	Smoothing, Centroiding, Background subtraction
MS2 verified by standard	No	Nomenclature for intact lipid molecule	Yes
Background check at MS1	No	Nomenclature for fragment ions	N/A
Background check at MS2	No	Further identification remarks	-

28) Cer[M+HCOO]- / Lipid quantification

Quantitative	No	Batch correction	No
Normalization to reference	No	Further quantification remarks	-

29) FA[M-H]- / Lipid identification

Lipid class	FA	Did you presume assumptions for identification?	No
MS Level for identification	MS1, MS2	Check on:	-
Identification level	Molecular species level	Limit of detection	S/N ratio > 3
Polarity mode	Negative	RT verified by standard	No
Type of negative (precursor)ion	[M-H]-	Separation of isobaric/isomeric interferece confirmed	No
Fragments for identification		Model for separation prediction	No

Fragment name
R1-H3O
R1-H-CO2

Isotope correction at MS1	No	Additional dimension/techniques	-
Isotope correction at MS2	No	Lipid Identification Software	LipidSearch
MS1 verified by standard	No	Data manipulation	Smoothing, Centroiding, Background subtraction
MS2 verified by standard	No	Nomenclature for intact lipid molecule	Yes
Background check at MS1	No	Nomenclature for fragment ions	N/A
Background check at MS2	No	Further identification remarks	-

29) FA[M-H]- / Lipid quantification

Quantitative	No	Batch correction	No
Normalization to reference	No	Further quantification remarks	-

30) HexCer[M+HCOO]- / Lipid identification

Lipid class	HexCer	Did you presume assumptions for identification?	No
MS Level for identification	MS1, MS2	Check on:	-
Identification level	Molecular species level	Limit of detection	S/N ratio > 3
Polarity mode	Negative	RT verified by standard	No
Type of negative (precursor)ion	[M+HCOO]-	Separation of isobaric/isomeric interferece confirmed	No
Fragments for identification		Model for separation prediction	No

Fragment name
-HG(Hex,180)
FA1(+C2H3N)
-HG(Hex,162)

Isotope correction at MS1	No	Additional dimension/techniques	-
Isotope correction at MS2	No	Lipid Identification Software	LipidSearch
MS1 verified by standard	No	Data manipulation	Smoothing, Centroiding, Background subtraction
MS2 verified by standard	No	Nomenclature for intact lipid molecule	Yes
Background check at MS1	No	Nomenclature for fragment ions	N/A
Background check at MS2	No	Further identification remarks	-

30) HexCer[M+HCOO]- / Lipid quantification

Quantitative	No	Batch correction	No
Normalization to reference	No	Further quantification remarks	-

31) LPC[M-H]- / Lipid identification

Lipid class	LPC	Did you presume assumptions for identification?	No
MS Level for identification	MS1, MS2	Check on:	-
Identification level	Molecular species level	Limit of detection	S/N ratio > 3
Polarity mode	Negative	RT verified by standard	No
Type of negative (precursor)ion	[M-H]-	Separation of isobaric/isomeric interferece confirmed	No
Fragments for identification		Model for separation prediction	No

Fragment name
HG(PC,224)
FA1(+O)

Isotope correction at MS1	No	Additional dimension/techniques	-
Isotope correction at MS2	No	Lipid Identification Software	LipidSearch
MS1 verified by standard	No	Data manipulation	Smoothing, Centroiding, Background subtraction
MS2 verified by standard	No	Nomenclature for intact lipid molecule	Yes
Background check at MS1	Yes	Nomenclature for fragment ions	N/A
Background check at MS2	No	Further identification remarks	-

31) LPC[M-H]- / Lipid quantification

Quantitative	No	Batch correction	No
Normalization to reference	No	Further quantification remarks	-

32) LPE[M-H]- / Lipid identification

Lipid class	LPE	Did you presume assumptions for identification?	No
MS Level for identification	MS1, MS2	Check on:	-
Identification level	Molecular species level	Limit of detection	S/N ratio > 3
Polarity mode	Negative	RT verified by standard	No
Type of negative (precursor)ion	[M-H]-	Separation of isobaric/isomeric interferece confirmed	No
Fragments for identification		Model for separation prediction	No

Fragment name
-FA1(-H)
GP(153)
FA1(+O)

Isotope correction at MS1	No	Additional dimension/techniques	-
Isotope correction at MS2	No	Lipid Identification Software	LipidSearch
MS1 verified by standard	No	Data manipulation	Smoothing, Centroiding, Background subtraction
MS2 verified by standard	No	Nomenclature for intact lipid molecule	No
Background check at MS1	Yes	Nomenclature for fragment ions	N/A
Background check at MS2	No	Further identification remarks	-

32) LPE[M-H]- / Lipid quantification

Quantitative	No	Batch correction	No
Normalization to reference	No	Further quantification remarks	-

33) PC[M+HCOO]- / Lipid identification

Lipid class	PC	Did you presume assumptions for identification?	No
MS Level for identification	MS1, MS2	Check on:	-
Identification level	Molecular species level	Limit of detection	S/N ratio > 3
Polarity mode	Negative	RT verified by standard	No
Type of negative (precursor)ion	[M+HCOO]-	Separation of isobaric/isomeric interferece confirmed	No
Fragments for identification		Model for separation prediction	No

Fragment name
HG(PC,168)
FA1(+O)
FA2(+O)

Isotope correction at MS1	No	Additional dimension/techniques	-
Isotope correction at MS2	No	Lipid Identification Software	LipidSearch
MS1 verified by standard	No	Data manipulation	Smoothing, Centroiding, Background subtraction
MS2 verified by standard	No	Nomenclature for intact lipid molecule	No
Background check at MS1	Yes	Nomenclature for fragment ions	N/A
Background check at MS2	No	Further identification remarks	-

33) PC[M+HCOO]- / Lipid quantification

Quantitative	No	Batch correction	No
Normalization to reference	No	Further quantification remarks	-

34) LPI[M-H]- / Lipid identification

Lipid class	LPI	Did you presume assumptions for identification?	No
MS Level for identification	MS1, MS2	Check on:	-
Identification level	Molecular species level	Limit of detection	S/N ratio > 3
Polarity mode	Negative	RT verified by standard	No
Type of negative (precursor)ion	[M-H]-	Separation of isobaric/isomeric interferece confirmed	No
Fragments for identification		Model for separation prediction	No

Fragment name
-(C6H12O6, 180)
GP(153)
HG(PI,241)
-FA1(+HO)
FA1(+O)

Isotope correction at MS1	No	Additional dimension/techniques	-
Isotope correction at MS2	No	Lipid Identification Software	LipidSearch
MS1 verified by standard	No	Data manipulation	Smoothing, Centroiding, Background subtraction
MS2 verified by standard	No	Nomenclature for intact lipid molecule	No
Background check at MS1	Yes	Nomenclature for fragment ions	N/A
Background check at MS2	No	Further identification remarks	-

34) LPI[M-H]- / Lipid quantification

Quantitative	No	Batch correction	No
Normalization to reference	No	Further quantification remarks	-

35) LPG[M-H]- / Lipid identification

Lipid class	LPG	Did you presume assumptions for identification?	No
MS Level for identification	MS1, MS2	Check on:	-
Identification level	Molecular species level	Limit of detection	S/N ratio > 3
Polarity mode	Negative	RT verified by standard	No
Type of negative (precursor)ion	[M-H]-	Separation of isobaric/isomeric interferece confirmed	No
Fragments for identification		Model for separation prediction	No

Fragment name
GP(153)
-FA1(-H)
P(79)
FA1(+O)

Isotope correction at MS1	No	Additional dimension/techniques	-
Isotope correction at MS2	No	Lipid Identification Software	LipidSearch
MS1 verified by standard	No	Data manipulation	Smoothing, Centroiding, Background subtraction
MS2 verified by standard	No	Nomenclature for intact lipid molecule	No
Background check at MS1	Yes	Nomenclature for fragment ions	N/A
Background check at MS2	No	Further identification remarks	-

35) LPG[M-H]- / Lipid quantification

Quantitative	No	Batch correction	No
Normalization to reference	No	Further quantification remarks	-

36) LPS[M-H]- / Lipid identification

Lipid class	LPS	Did you presume assumptions for identification?	No
MS Level for identification	MS1, MS2	Check on:	-
Identification level	Molecular species level	Limit of detection	S/N ratio > 3
Polarity mode	Negative	RT verified by standard	No
Type of negative (precursor)ion	[M-H]-	Separation of isobaric/isomeric interferece confirmed	No
Fragments for identification		Model for separation prediction	No

Fragment name
GP(153)
P(79)
P(97)
FA1(+O)
-(C3H5NO2,87)

Isotope correction at MS1	No	Additional dimension/techniques	-
Isotope correction at MS2	No	Lipid Identification Software	LipidSearch
MS1 verified by standard	No	Data manipulation	Smoothing, Centroiding, Background subtraction
MS2 verified by standard	No	Nomenclature for intact lipid molecule	No
Background check at MS1	Yes	Nomenclature for fragment ions	N/A
Background check at MS2	No	Further identification remarks	-

36) LPS[M-H]- / Lipid quantification

Quantitative	No	Batch correction	No
Normalization to reference	No	Further quantification remarks	-

37) PC P[M+HCOO]- / Lipid identification

Lipid class	PC P	Did you presume assumptions for identification?	No
MS Level for identification	MS1, MS2	Check on:	-
Identification level	Molecular species level	Limit of detection	S/N ratio > 3
Polarity mode	Negative	RT verified by standard	No
Type of negative (precursor)ion	[M+HCOO]-	Separation of isobaric/isomeric interferece confirmed	No
Fragments for identification		Model for separation prediction	No

Fragment name
HG(PC)-(CH3+HCOO)
-(CH3+HCOO)
FA2(+O)
FA1

Isotope correction at MS1	No	Additional dimension/techniques	-
Isotope correction at MS2	No	Lipid Identification Software	LipidSearch
MS1 verified by standard	No	Data manipulation	Smoothing, Centroiding, Background subtraction
MS2 verified by standard	No	Nomenclature for intact lipid molecule	No
Background check at MS1	Yes	Nomenclature for fragment ions	N/A
Background check at MS2	No	Further identification remarks	-

37) PC P[M+HCOO]- / Lipid quantification

Quantitative	No	Batch correction	No
Normalization to reference	No	Further quantification remarks	-

38) PC O[M+HCOO]- / Lipid identification

Lipid class	PC O	Did you presume assumptions for identification?	No
MS Level for identification	MS1, MS2	Check on:	-
Identification level	Molecular species level	Limit of detection	S/N ratio > 3
Polarity mode	Negative	RT verified by standard	No
Type of negative (precursor)ion	[M+HCOO]-	Separation of isobaric/isomeric interferece confirmed	No
Fragments for identification		Model for separation prediction	No

Fragment name
HG(PC)-(CH3+HCOO)
-(CH3+HCOO)
-FA2(-H)
FA2(+O)
FA1

Isotope correction at MS1	No	Additional dimension/techniques	-
Isotope correction at MS2	No	Lipid Identification Software	LipidSearch
MS1 verified by standard	No	Data manipulation	Smoothing, Centroiding, Background subtraction
MS2 verified by standard	No	Nomenclature for intact lipid molecule	No
Background check at MS1	Yes	Nomenclature for fragment ions	N/A
Background check at MS2	No	Further identification remarks	-

38) PC O[M+HCOO]- / Lipid quantification

Quantitative	No	Batch correction	No
Normalization to reference	No	Further quantification remarks	-

39) PE[M-H]- / Lipid identification

Lipid class	PE	Did you presume assumptions for identification?	No
MS Level for identification	MS1, MS2	Check on:	-
Identification level	Molecular species level	Limit of detection	S/N ratio > 3
Polarity mode	Negative	RT verified by standard	No
Type of negative (precursor)ion	[M-H]-	Separation of isobaric/isomeric interferece confirmed	No
Fragments for identification		Model for separation prediction	No

Fragment name
HG(PE,196)
GP(153)
HG(PE,140)
FA1(+O)
FA2(+O)
-FA1(-H)
-FA2(-H)

Isotope correction at MS1	No	Additional dimension/techniques	-
Isotope correction at MS2	No	Lipid Identification Software	LipidSearch
MS1 verified by standard	No	Data manipulation	Smoothing, Centroiding, Background subtraction
MS2 verified by standard	No	Nomenclature for intact lipid molecule	No
Background check at MS1	Yes	Nomenclature for fragment ions	N/A
Background check at MS2	No	Further identification remarks	-

39) PE[M-H]- / Lipid quantification

Quantitative	No	Batch correction	No
Normalization to reference	No	Further quantification remarks	-

40) PE O[M-H]- / Lipid identification

Lipid class	PE O	Did you presume assumptions for identification?	No
MS Level for identification	MS1, MS2	Check on:	-
Identification level	Molecular species level	Limit of detection	S/N ratio $>$ 3
Polarity mode	Negative	RT verified by standard	No
Type of negative (precursor)ion	[M-H]-	Separation of isobaric/isomeric interferece confirmed	No
Fragments for identification		Model for separation prediction	No

Fragment name
GP(153)
FA2(+O)
-FA2(-H)
GP(135)

Isotope correction at MS1	No	Additional dimension/techniques	-
Isotope correction at MS2	No	Lipid Identification Software	LipidSearch
MS1 verified by standard	No	Data manipulation	Smoothing, Centroiding, Background subtraction
MS2 verified by standard	No	Nomenclature for intact lipid molecule	No
Background check at MS1	Yes	Nomenclature for fragment ions	N/A
Background check at MS2	No	Further identification remarks	-

40) PE O[M-H]- / Lipid quantification

Quantitative	No	Batch correction	No
Normalization to reference	No	Further quantification remarks	-

41) PG[M-H]- / Lipid identification

Lipid class	PG	Did you presume assumptions for identification?	No
MS Level for identification	MS1, MS2	Check on:	-
Identification level	Molecular species level	Limit of detection	S/N ratio > 3
Polarity mode	Negative	RT verified by standard	No
Type of negative (precursor)ion	[M-H]-	Separation of isobaric/isomeric interferece confirmed	No
Fragments for identification		Model for separation prediction	No

Fragment name
-FA1(-H)
-FA2(-H)
FA1(+O)
FA2(+O)
HG(PG,227)
HG(PG,171)

Isotope correction at MS1	No	Additional dimension/techniques	-
Isotope correction at MS2	No	Lipid Identification Software	LipidSearch
MS1 verified by standard	No	Data manipulation	Smoothing, Centroiding, Background subtraction
MS2 verified by standard	No	Nomenclature for intact lipid molecule	No
Background check at MS1	Yes	Nomenclature for fragment ions	N/A
Background check at MS2	No	Further identification remarks	-

41) PG[M-H]- / Lipid quantification

Quantitative	No	Batch correction	No
Normalization to reference	No	Further quantification remarks	-

42) PI[M-H]- / Lipid identification

Lipid class	PI	Did you presume assumptions for identification?	No
MS Level for identification	MS1, MS2	Check on:	-
Identification level	Molecular species level	Limit of detection	S/N ratio > 3
Polarity mode	Negative	RT verified by standard	No
Type of negative (precursor)ion	[M-H]-	Separation of isobaric/isomeric interferece confirmed	No
Fragments for identification		Model for separation prediction	No

Fragment name
-FA1(-H)
FA1(+O)
-FA2(-H)
HG(PI,241)
FA2(+O)
GP(153)

Isotope correction at MS1	No	Additional dimension/techniques	-
Isotope correction at MS2	No	Lipid Identification Software	LipidSearch
MS1 verified by standard	No	Data manipulation	Smoothing, Centroiding, Background subtraction
MS2 verified by standard	No	Nomenclature for intact lipid molecule	No
Background check at MS1	Yes	Nomenclature for fragment ions	N/A
Background check at MS2	No	Further identification remarks	-

42) PI[M-H]- / Lipid quantification

Quantitative	No	Batch correction	No
Normalization to reference	No	Further quantification remarks	-

43) PS[M-H]- / Lipid identification

Lipid class	PS	Did you presume assumptions for identification?	No
MS Level for identification	MS1, MS2	Check on:	-
Identification level	Molecular species level	Limit of detection	S/N ratio > 3
Polarity mode	Negative	RT verified by standard	No
Type of negative (precursor)ion	[M-H]-	Separation of isobaric/isomeric interferece confirmed	No
Fragments for identification		Model for separation prediction	No

Fragment name
-FA1(-H)-(C3H5NO2)
-FA2(-H)-(C3H5NO2)
FA1(+O)
FA2(+O)
GP(153)
-(C3H5NO2,87)

Isotope correction at MS1	No	Additional dimension/techniques	-
Isotope correction at MS2	No	Lipid Identification Software	LipidSearch
MS1 verified by standard	No	Data manipulation	Smoothing, Centroiding, Background subtraction
MS2 verified by standard	No	Nomenclature for intact lipid molecule	No
Background check at MS1	Yes	Nomenclature for fragment ions	N/A
Background check at MS2	No	Further identification remarks	-

43) PS[M-H]- / Lipid quantification

Quantitative	No	Batch correction	No
Normalization to reference	No	Further quantification remarks	-

44) SM[M+HCOO]- / Lipid identification

Lipid class	SM	Did you presume assumptions for identification?	No
MS Level for identification	MS1, MS2	Check on:	-
Identification level	Molecular species level	Limit of detection	S/N ratio > 3
Polarity mode	Negative	RT verified by standard	No
Type of negative (precursor)ion	[M+HCOO]-	Separation of isobaric/isomeric interferece confirmed	No
Fragments for identification		Model for separation prediction	No

Fragment name
HG(PC,168)
FA1(+O)
-(CH3)

Isotope correction at MS1	No	Additional dimension/techniques	-
Isotope correction at MS2	No	Lipid Identification Software	LipidSearch
MS1 verified by standard	No	Data manipulation	Smoothing, Centroiding, Background subtraction
MS2 verified by standard	No	Nomenclature for intact lipid molecule	No
Background check at MS1	Yes	Nomenclature for fragment ions	N/A
Background check at MS2	No	Further identification remarks	-

44) SM[M+HCOO]- / Lipid quantification

Quantitative	No	Batch correction	No
Normalization to reference	No	Further quantification remarks	-
