# Supplementary material for: Mass spectrometry-based characterisation of the cardiac microtissue metabolome and lipidome
Source: Metabolomics. 2025 Apr 21;21(3):54. doi: 10.1007/s11306-025-02252-0 (PMC12011886; doi:10.1007/s11306-025-02252-0)
Supplement: Supplementary file 3 — Supplementary Material 3 [file 11306_2025_2252_MOESM3_ESM.docx]

Supplementary Information

Mass spectrometry-based characterisation of the cardiac microtissue metabolome and lipidome

Tara J. Bowen^1,†^; Andrew R. Hall^2^; Andrew D. Southam^1,3^; Ossama Edbali^1,3^; Ralf J. M. Weber^1,3^; Amanda Wilson^4^; Amy Pointon^2^; Mark R. Viant^1,3,*^

^1^ School of Biosciences, University of Birmingham, Edgbaston, Birmingham B15 2TT, UK

^2^ Safety Sciences, Clinical Pharmacology and Safety Sciences, BioPharmaceuticals R&D, AstraZeneca, Cambridge, UK

^3^ Phenome Centre Birmingham, University of Birmingham, Edgbaston, Birmingham B15 2TT, UK

^4^ Integrated Bioanalysis, Clinical Pharmacology and Safety Sciences, BioPharmaceuticals R&D, AstraZeneca, Cambridge, UK

^†^ Current affiliation: Medicines Discovery Catapult, Alderley Park, Cheshire, SK10 4TG, UK

*Correspondence: [m.viant@bham.ac.uk](mailto:m.viant@bham.ac.uk)

# Supplementary Methods

## Cell culture

Three cell types, mainly, (1) human induced pluripotent stem cell derived cardiomyocytes (hiPSC-CMs), differentiated from blood-derived iPSCs from a healthy Caucasian female, aged 30-39 (FUJIFILM Cellular Dynamics, Inc., Cat. No. 11713), (2) primary human cardiac fibroblasts (hCFs), isolated from heart tissue of a healthy 46-year-old Caucasian male (Lonza, Cat. No. CC-2904) and (3) primary human microvascular endothelial cells (HCMECs), isolated from the ventricle of a 30-year-old male (Lonza, Cat. No. CC-7030), were cultured as per manufacturers’ instructions for monolayer cultures. All commercial suppliers of cells used in this study have respective ethical and donor consent forms for cell line generation, and subsequent use in Research and Development.

Cardiac microtissues were cultured as per Archer *et al.* (2018). Briefly, cardiac microtissues were seeded in ultra-low attachment 384-well plates (*ca.* 500 cells/well in a 4:2:1 ratio of hiPSC-CMs/hCFs/hCMECs) in 40 µL of 50/50 iCell plating media/hCMEC media. 40 µL media was added after 48-hours, and then media changed every 3 to 4 days. After 14-days of culture, cardiac microtissues had formed and were ready for experimentation.

All experiments were approved and conducted in line with AstraZeneca ethics and Human Biological Sample tissue handling where appropriate.

## Microtissue sample generation

Samples of 154-pooled microtissues were generated by aspiration of 14-day mature microtissues and their culture media from 154 neighbouring wells of a 384-well ultra-low adhesion U-bottom microplate and subsequently dispensed onto the filter surface of a cell strainer (37 μm reversible strainer, StemCell Technologies). Samples were washed with 3x 1.0 mL 0.9% sodium chloride, followed by 1.0 mL ultrapure (18.2 mQ) water. Suction was applied to ensure removal of any residual wash solution before rapid freezing of the microtissue-containing cell strainer in dry ice/ethanol bath (Bowen *et al.*, 2021). Samples were kept on dry ice during collection before being transferred to a freezer maintained at -80°C. Samples were later shipped on dry ice then returned to -80°C storage conditions until preparation for metabolomics analyses. Process blanks were generated by sampling of media only (no microtissues) by the same approach.

Polar metabolites were extracted from 15 of the samples (28-pooled microtissues each) by addition of 200 μL 4:1 (v/v) methanol/water (Bowen *et al.*, 2021). After centrifugation (20,000-*g* for 20-minutes at 4°C), a single pooled sample was created by combining 180 μL supernatant from each of the 15 samples. The pooled sample was vortexed, then split between four microcentrifuge tubes, 490 μL in each (equivalent to *ca.* 377 cardiac microtissues, or *ca.* 1.9x10^5^ cells) before drying in a SpeedVac concentrator (Savant SPD111V, Thermo Scientific). Dried extracts were stored at -80°C. On the day of analysis, dried polar extracts were resuspended in 38 μL 1.5:1.5:1.0 (v/v/v) acetonitrile/methanol/water. Each resuspended sample was vortexed for 30-seconds then centrifuged at 20,000*-g* for 20-minutes at 4°C.

Intracellular lipids were extracted from 15 of the samples by addition of 240 μL 2:1 (v/v) chloroform/methanol (Bowen *et al.*, 2021). After centrifugation (4,000-*g* for 20-minutes at 4°C), 200 μL supernatant from each sample was combined to form a single pooled sample which was subsequently vortexed then split between four 250 μL glass micro-inserts held in 1.75 mL glass vials, 590 μL in each (equivalent to *ca.* 379 cardiac microtissues, or *ca.* 1.9x10^5^ cells). Aliquots were dried under N_2_ (Techne Dri-Block DB100/3 sample concentrator), then stored at -80°C. On the day of analysis, dried lipid extracts were resuspended in 38 μL 3:1 (v/v) isopropanol/water, vortexed, and centrifuged at 4,000*-g* for 20-minutes at 4°C. 33 μL of supernatant from each resuspended extract was transferred to a separate 300 μL polyspring glass insert within an LC vial for analysis.

Process blanks were prepared as described for samples. Extracts from three separate process blank samples were pooled to form a single dried extract.

## Data acquisition by UHPLC-MS

### HILIC UHPLC-MS

Chromatographic separation was performed using an Accucore 150 Amide column (100 x 2.1 mm, 2.6 μm, Thermo Scientific) with a pre-column UHPLC filter (2.1 mm ID x 0.2 μm filter cartridge, Thermo Scientific). Mobile phase A was 95% acetonitrile/water (10 mM ammonium formate, 0.1% formic acid), and mobile phase B was 50% acetonitrile/water (10 mM ammonium formate, 0.1% formic acid) for the positive ionisation mode. For the negative ionisation mode, mobile phase modifiers were 10 mM ammonium acetate and 0.1% acetic acid, in place of ammonium formate and formic acid, respectively. The gradient was as follows: t = 0.0, 1% B; t = 2.1, 1% B; t = 4.1, 15% B; t = 7.1, 50% B; t = 10.1, 95% B; t = 11.0, 95% B; t = 11.5, 1% B; t = 15.0, 1% B. All changes were linear (curve = 5). The flow rate was 0.4 mL/min, and the column temperature was 35°C. Mass spectrometry analysis was performed in positive and negative ionisation modes separately at a resolution of 120,000, between 70 and 1050 *m/z*. The Automatic Gain Control (AGC) target was 1x10^5^ and maximum injection time was set at 50 ms. Ion source parameters are detailed in Table S1. The sample injection volume was 2 μL.

***Table S1*** *Ion source and ion optics parameters for data acquisition by UHPLC-MS^n^*

| **Assay** | **Sheath Gas (Arb)** | **Aux gas (Arb)** | **Sweep gas (Arb)** | **Spray voltage (kV)** | **Ion transfer tube temperature (°C)** | **Vaporizer temperature (°C)** | **RF lens voltage (%)** |
| --- | --- | --- | --- | --- | --- | --- | --- |
| HILIC positive | 40 | 8 | 1 | 3.2 | 300 | 350 | 30 |
| HILIC negative | 40 | 8 | 1 | 2.7 | 300 | 350 | 30 |
| RP-C_30_ positive | 40 | 8 | 1 | 3.2 | 300 | 350 | 70 |
| RP-C_30_ negative | 40 | 8 | 1 | 2.7 | 300 | 350 | 70 |

An AcquireX Deep Scan (Thermo Scientific) workflow was implemented for the acquisition of MS^n^ fragmentation data. Three cycles of a sequence were executed, the sequence being composed of: two full scan injections of a process blank, the second of which was used to generate an exclusion list; a full scan injection of a column equilibration sample; a full scan injection of a study sample, used to modify the user-generated inclusion list of protonated or de-protonated ion forms of toxicologically relevant metabolites (MTox700+ biomarker list (Sostare *et al.*, 2022)); and five iterative injections for MS^n^ acquisition using the inclusion and exclusion lists, which were modified by AcquireX software after each injection, removing features from the inclusion list and adding them to the exclusion list if they had been used as a precursor ion. Injections of study sample within a cycle were from the same vial, with a different vial used for each cycle. Regarding acquisition settings of MS^n^ data: MS^1^ and higher-energy collisional dissociation (HCD) MS/MS (MS^2^) data were acquired by the Orbitrap; collision-induced dissociation (CID) MS/MS/MS (MS^3^) data were acquired for the three most intense fragments measured in the HCD spectra by the ion trap (Fig. S1).


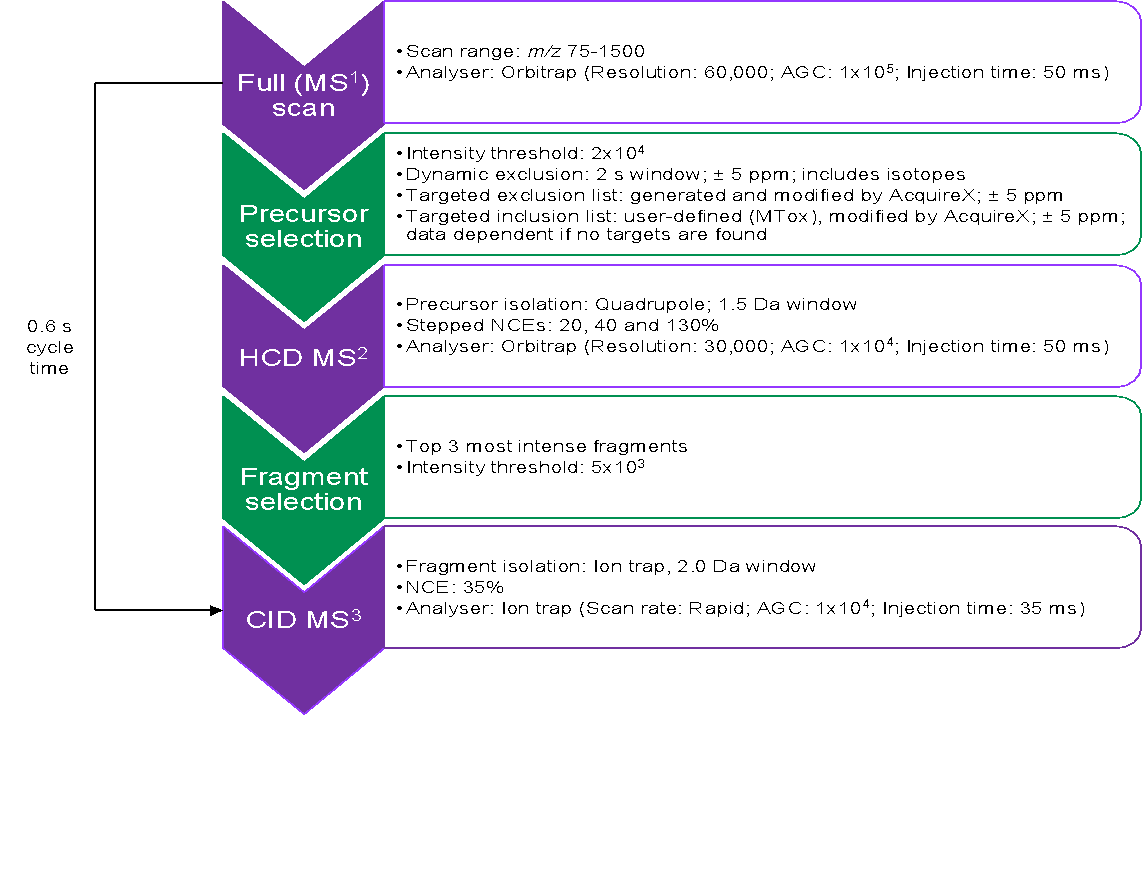


***Fig. S1*** *Flow chart describing MS^n^ data acquisition method when coupled to HILIC chromatography. Each scan cycle begins with full (MS^1^) scan by the Orbitrap, measurements from which are used to select precursors which meet defined criteria for subsequent fragmentation by higher-energy collisional dissociation (HCD) at three stepped normalised collision energies (NCEs). The resulting MS^2^ fragments are detected in the Orbitrap. Subsequent collision-induced dissociation (CID) is performed in the high-pressure cell of the dual-pressure linear ion trap on the three most intense MS^2^ fragments, provided they pass the defined intensity filter. The resulting MS^3^ fragments are detected in the low-pressure cell of the dual-pressure linear ion trap. The MS^2^ and subsequent MS^3^ is repeated for as many MS^1^ precursors as possible within the 0.6 s cycle time, before proceeding to the next MS^1^ scan, at which point the cycle repeats.*

### C_30_-based reversed phase UHPLC-MS

Chromatographic separation was performed on an Accucore C_30_ column (2.1 x 150 mm, 2.6 μm, Thermo Scientific), maintained at 55°C. Mobile phases A and B were 60:40 acetonitrile/water (20 mM ammonium formate) and 85.5:9.5:5 isopropanol/acetonitrile/water (20 mM ammonium formate). A 30-minute gradient elution was applied as follows: t = 0.0, 20% B; t = 2.5, 20% B; t = 2.6, 55% B; t = 12.0, 60% B; t = 12.1, 80% B; t = 19.0, 90% B; t = 21.0, 100% B; t = 23.0, 100% B; t = 23.1, 20% B, with all changes linear (curve = 5). The flow rate was 0.4 mL/min and the injection volume was 2 μL.

Mass spectrometry data were acquired in positive and negative ionisation modes separately, over a mass range of 150-2000 *m/z*, at a resolution of 120,000. The AGC target was 4x10^5^ and maximum injection time set at 50 ms. Ion source and ion optics settings are detailed in Table S1.

AcquireX was implemented for acquisition of MS^n^ data as described for the HILIC method. Regarding data acquisition settings: MS^1^ and HCD MS^2^ data were acquired in the Orbitrap; subsequent CID MS^2^, triggered by detection of a fragment ion at *m/*z 184.0733 or *m/*z 168.0431 in positive and negative ionisation, respectively, or CID MS^3^, triggered by detection of fragment ions formed as a result of one of 32 targeted neutral losses, were measured in the Orbitrap (Fig. S2).


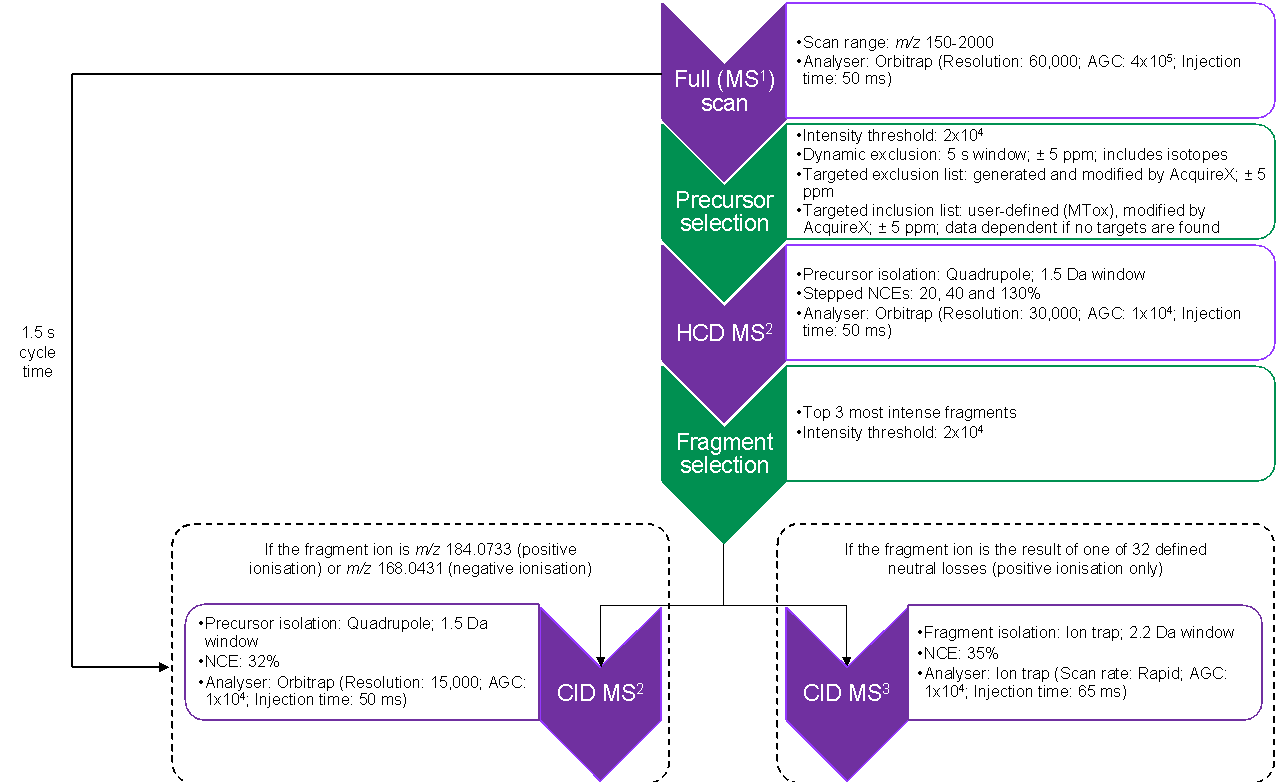


***Fig. S2*** *Flow chart describing MS^n^ data acquisition when coupled to RP-C_30_ chromatography. Each scan cycle begins with a full (MS^1^) scan by the Orbitrap. The MS^1^ measurements are used to select precursors which meet defined criteria for subsequent fragmentation by higher-energy collisional dissociation (HCD), at three stepped normalised collision energies (NCEs). The resulting MS^2^ fragments are detected in the Orbitrap. Subsequent collision-induced dissociation (CID) is performed in the high-pressure cell of the dual-pressure linear ion trap on the same precursor (MS^1^) ion if a fragment at m/z 184.0733 or m/z 168.0431 is detected in the HCD spectrum in positive and negative ionisation, respectively. These fragment ions correspond to the phosphocholine head group (protonation or de-methylation, respectively) of phosphatidylcholines, lyso-phosphatidylcholines and sphingomyelins. Alternatively, collision-induced dissociation (CID) may be performed on the three most intense MS^2^ fragments if they are formed by a defined neutral loss from the precursor ion. The defined neutral losses correspond to the loss of fatty acyls, typically observed in MS^2^ of triacylglycerols. The resulting MS^2^ or MS^3^ fragments are detected in the low-pressure cell of the dual-pressure linear ion trap. The HCD MS^2^ and subsequent CID MS^2^ or MS^3^ is repeated for as many MS^1^ precursors as possible within the 1.5 s cycle time, before proceeding to the next MS^1^ scan, at which point the cycle repeats.*

## Data processing and annotation

***Table*** ***S2*** *XCMS (Smith et al., 2006) parameters used for full scan (MS^1^) peak picking and grouping using mzML files generated by conversion of vendor format raw data files (.RAW) using ProteoWizard (v3.0.21215) (Chambers et al., 2012).*

| **Function** | **Parameter** | **HILIC chromatography** | **RP-C_30_ chromatography** |
| --- | --- | --- | --- |
| Peak detection – centwave algorithm | ppm | 7.5 | 5 |
|  | peakwidth | 5,40 | 5,40 |
|  | snthresh | 10 | 10 |
|  | prefilter | 3,100 | 3,100 |
|  | mzCenterFun | wMean | wMean |
|  | mzdiff | 0.001 | 0.001 |
| Peak grouping - PeakDensity | bw | 0.25 | 0.25 |
|  | Minfrac | 0.5 | 0.5 |
|  | binSize | 0.001 | 0.01 |
|  | maxFeatures | 50 | 50 |

***Table S3*** *Compound Discoverer™ (v3.3, Thermo Scientific) workflow processing nodes and parameters.*

| **Node** | **Parameter** | **Value** |
| --- | --- | --- |
| Detect peaks | SNR threshold | 1.5 |
| Align retention times | Function | Adaptive curve model |
|  | Mass tolerance | 5 ppm |
|  | Maximum shift | 2 minutes |
| Detect compounds | Mass tolerance | 5 ppm |
|  | Minimum peak intensity | 1x10^4^ |
|  | Minimum scans per peak | 5 |
|  | Chromatographic SNR threshold | 1.5 |
|  | Maximum peak width | 1 minute |
|  | Gap ratio threshold | 0.35 |
|  | Isotope pattern detection | Default |
|  | Ion forms | All available, except ‘3M’ species |
|  | Base ions | [M+H]^+^/[M-H]^-^ |
|  | Remove singlets | True |
| Group compounds | Reported intensity | Use intensity (peak area integration) from most common ion |
|  | Mass tolerance | 5 ppm |
|  | Retention time tolerance | 0.2 minutes |
|  | Peak rating contributions | Default |
| Mark background compounds | Maximum sample/blank ratio | 5 |
| Search mzCloud | Search MS^n^ tree | True |
|  | Precursor mass tolerance | 5 ppm |
|  | Fragment mass tolerance FT | 10 ppm |
|  | Fragment mass tolerance IT | 0.4 Da |
|  | Search space | Autoprocessed and reference libraries |
|  | Spectral match scoring metric | HighChem HighRes |
|  | Spectral match score threshold | 0.6 |
| Search mzVault | Precursor mass tolerance | 5 ppm |
|  | Fragment mass tolerance FT | 10 ppm |
|  | Fragment mass tolerance IT | 0.4 Da |
|  | Search space | Libraries contributed by Prof. Bamba (Kyushu University); LipidBlast spectral database |
|  | Spectral match scoring metric | HighChem HighRes |
|  | Spectral match score threshold | 0.6 |
| Assign compound annotations | Mass tolerance | 5 ppm |

***Table S4*** *LipidSearch™ (v5.0, Thermo Scientific) workflow for processing and annotating data acquired by RP-C_30_ UHPLC-MS.*

| **Step** | **Parameter** | **Value** |
| --- | --- | --- |
| Peak detection | Peak detection | Default |
| Lipid annotation | Precursor mass tolerance | 5 ppm |
|  | Product ion mass tolerance | 8 ppm |
|  | Product ion intensity threshold | 5 |
|  | Reference list | All lipid classes, substituents and adduct ion definitions in the LipidSearch™ *in silico* database |
| Sample alignment | Retention time tolerance | 0.2 minutes |
|  | SNR threshold^^[[1]](#footnote-1)^^ | 3 |
|  | IR threshold^^[[2]](#footnote-2)^^ | 1.5 |
|  | VPR threshold^^[[3]](#footnote-3)^^ | 0.5 |

***Table S5*** *LipidSearch™ (v5.0, Thermo Scientific) annotation grade definitions. The annotation grades are defined based on the degree of MS/MS product ions assigned to the lipid molecule.*

| **Grade** | **Definition** |
| --- | --- |
| A | All of the class-specific ions and substituent-specific ions that specifies the structure are assigned. Full assignment of the block structure. |
| B | One of the class-specific ions and substituent-specific ions that specifies the structure. Partial assignment of the block structure. |
| C | Either a class-specific ion or a substituent-specific ion that specifies the structure is assigned. Only Markush structures can be assigned. |
| D | A product ion that specifies the structure is not assigned. Only Markush structure assignment is possible. |

Manual inspection and curation of automatically assigned lipid annotations were conducted based on the following criteria:

- Only LipidSearch assignments of at least grade C were retained.
- Only lipid species with annotations based on the presence of at least two signature fragment ions in the corresponding MS/MS spectra were retained. Example MS/MS spectra for each lipid class are shown in Fig. S3 and indicate signature MS/MS fragments for each lipid class in the ionisation mode(s) in which they were measured.
- Lipid species unlikely to be present within the biological sample based on public knowledgebase were excluded, e.g., rejection of lipids with one or more fatty acyl chains containing >26 carbons or >6 double bonds.
- Exclusion of lipid species with retention time outside of the expected window according to their lipid class (Jankevics et al., 2021) and outliers to the intra-class retention time trend in terms of number of carbons and double bonds in fatty acyl chains, i.e., linear increase in retention time with increasing carbon chain length and linear decrease in retention time with increasing unsaturation of carbon chains.

# Supplementary Results

***Table S6*** *Assessment of the analytical sensitivity and MS^n^ coverage of UHPLC-MS^n^ analyses of intracellular extracts from cardiac microtissues. The table presents the number of features or compounds extracted from the raw data by XCMS and Compound Discoverer, respectively, how many features or compounds remained after removal of blank features and features present in less than two of three technical replicates, and for how many features or compounds was at least MS^2^ fragmentation data measured.*

| **Processing software** | **Description of data reported** | **HILIC positive** | **HILIC negative** | **RP-C_30_ positive** | **RP-C_30_ negative** |
| --- | --- | --- | --- | --- | --- |
| XCMS & msPurity | Total number of features | 2504 | 2008 | 7454 | 7718 |
|  | Number of features following blank and replicate filtering | 1309 | 1387 | 4752 | 5406 |
|  | Number of features associated with MS^2^ data | 631 | 619 | 2942 | 2562 |
| Compound Discoverer | Total number of compounds | 4982 | 12142 | 35062 | 20289 |
|  | Number of compounds after blank and replicate filtering | 961 | 1709 | 10264 | 7955 |
|  | Number of compounds with MS^2^ data | 825 | 1520 | 9233 | 6531 |

***Table*** ***S8*** *Metabolic pathways represented by the curated list of polar metabolites. Listed are the names of KEGG biochemical pathways, the total number of metabolites within each pathway (‘Total’) and the number of metabolites within the curated list of polar metabolites detected in intracellular extracts of cardiac microtissues which mapped to each pathway (‘Hits’).*

| Pathway Name | Total | Hits |
| --- | --- | --- |
| Aminoacyl-tRNA biosynthesis | 48 | 18 |
| Purine metabolism | 65 | 16 |
| Alanine, aspartate and glutamate metabolism | 28 | 12 |
| Arginine biosynthesis | 14 | 11 |
| Amino sugar and nucleotide sugar metabolism | 37 | 11 |
| Pyrimidine metabolism | 39 | 11 |
| Arginine and proline metabolism | 38 | 10 |
| Glycine, serine and threonine metabolism | 33 | 9 |
| Glyoxylate and dicarboxylate metabolism | 32 | 8 |
| Cysteine and methionine metabolism | 33 | 8 |
| Glycerophospholipid metabolism | 36 | 8 |
| Pantothenate and CoA biosynthesis | 19 | 7 |
| Citrate cycle (TCA cycle) | 20 | 7 |
| Galactose metabolism | 27 | 7 |
| Glutathione metabolism | 28 | 7 |
| beta-Alanine metabolism | 21 | 6 |
| Glycolysis / Gluconeogenesis | 26 | 6 |
| Valine, leucine and isoleucine biosynthesis | 8 | 5 |
| Nicotinate and nicotinamide metabolism | 15 | 5 |
| Pentose phosphate pathway | 22 | 5 |
| Pyruvate metabolism | 22 | 5 |
| Lysine degradation | 25 | 5 |
| Inositol phosphate metabolism | 30 | 5 |
| Valine, leucine and isoleucine degradation | 40 | 5 |
| Tyrosine metabolism | 42 | 5 |
| Butanoate metabolism | 15 | 4 |
| Histidine metabolism | 16 | 4 |
| Starch and sucrose metabolism | 18 | 4 |
| Fructose and mannose metabolism | 20 | 4 |
| Sphingolipid metabolism | 21 | 4 |
| D-Glutamine and D-glutamate metabolism | 6 | 3 |
| Ascorbate and aldarate metabolism | 8 | 3 |
| Pentose and glucuronate interconversions | 18 | 3 |
| Primary bile acid biosynthesis | 46 | 3 |
| Phenylalanine, tyrosine and tryptophan biosynthesis | 4 | 2 |
| Synthesis and degradation of ketone bodies | 5 | 2 |
| Nitrogen metabolism | 6 | 2 |
| Taurine and hypotaurine metabolism | 8 | 2 |
| Vitamin B6 metabolism | 9 | 2 |
| Phenylalanine metabolism | 10 | 2 |
| Glycerolipid metabolism | 16 | 2 |
| Phosphatidylinositol signalling system | 28 | 2 |
| Porphyrin and chlorophyll metabolism | 30 | 2 |
| Tryptophan metabolism | 41 | 2 |
| Steroid biosynthesis | 42 | 2 |
| Neomycin, kanamycin and gentamicin biosynthesis | 2 | 1 |
| Linoleic acid metabolism | 5 | 1 |
| Thiamine metabolism | 7 | 1 |
| Ubiquinone and other terpenoid-quinone biosynthesis | 9 | 1 |
| One carbon pool by folate | 9 | 1 |
| Biotin metabolism | 10 | 1 |
| alpha-Linolenic acid metabolism | 13 | 1 |
| Ether lipid metabolism | 20 | 1 |
| Propanoate metabolism | 23 | 1 |
| Folate biosynthesis | 27 | 1 |
| Arachidonic acid metabolism | 36 | 1 |
| Fatty acid degradation | 39 | 1 |


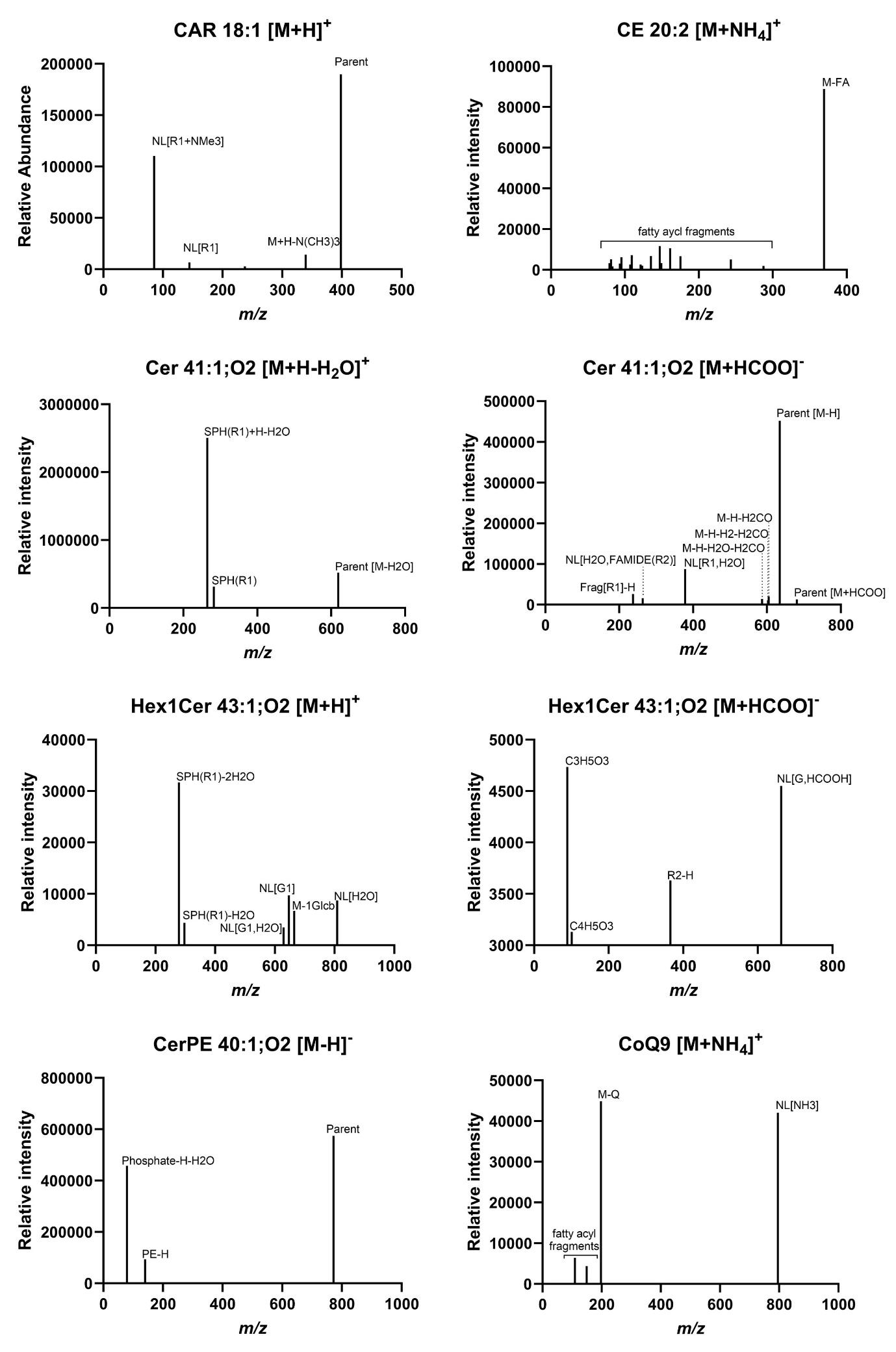


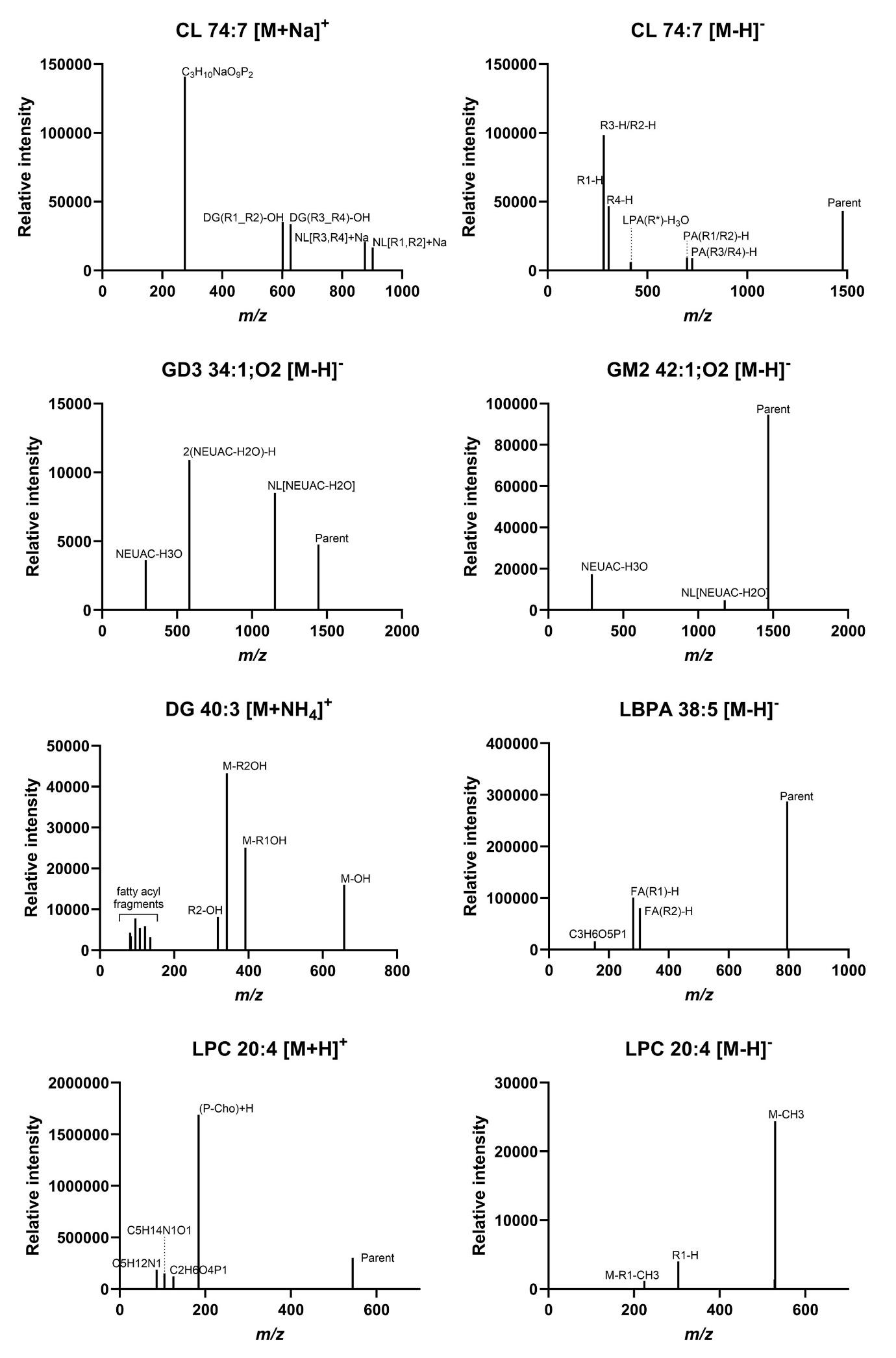

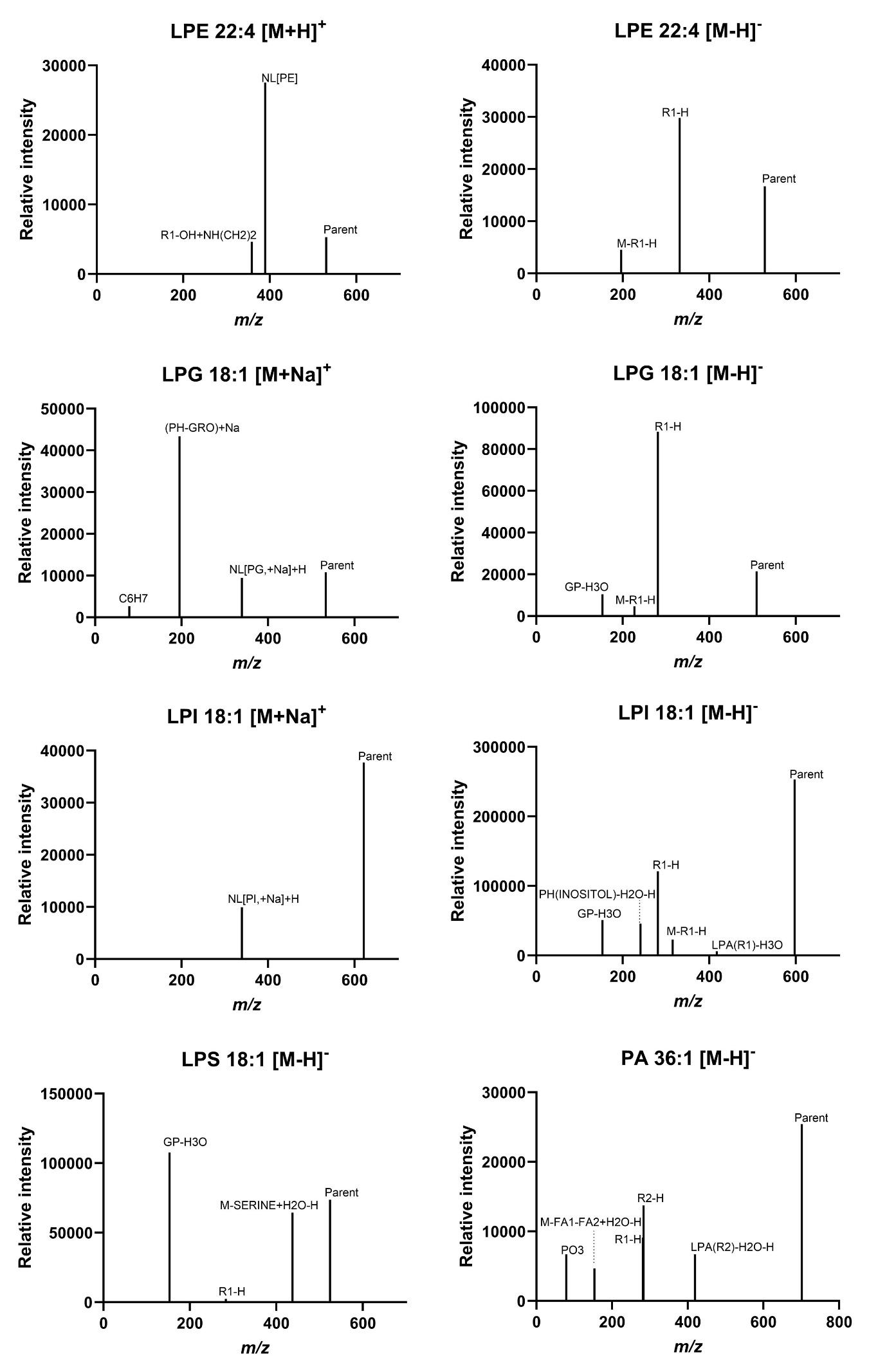

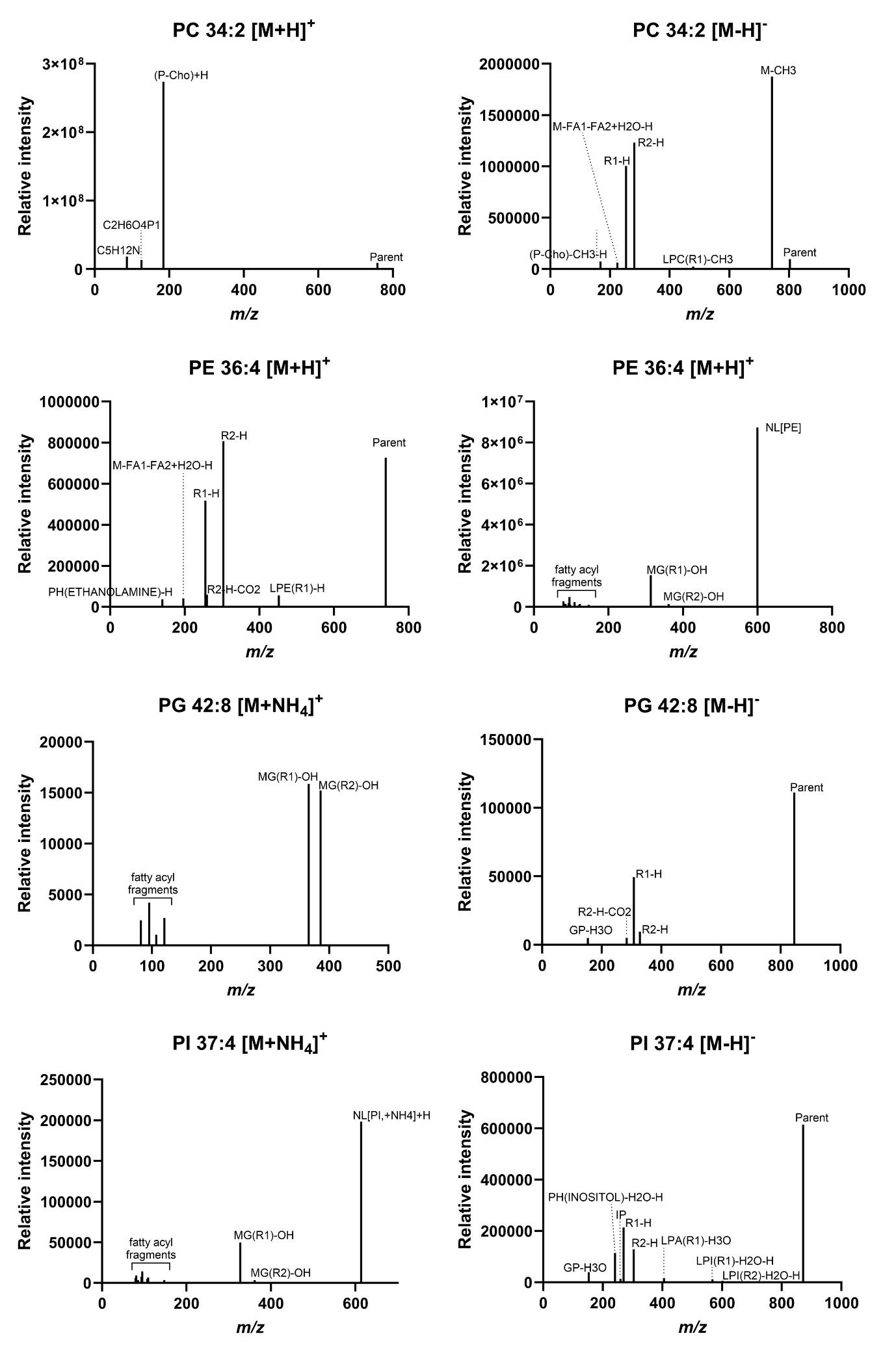

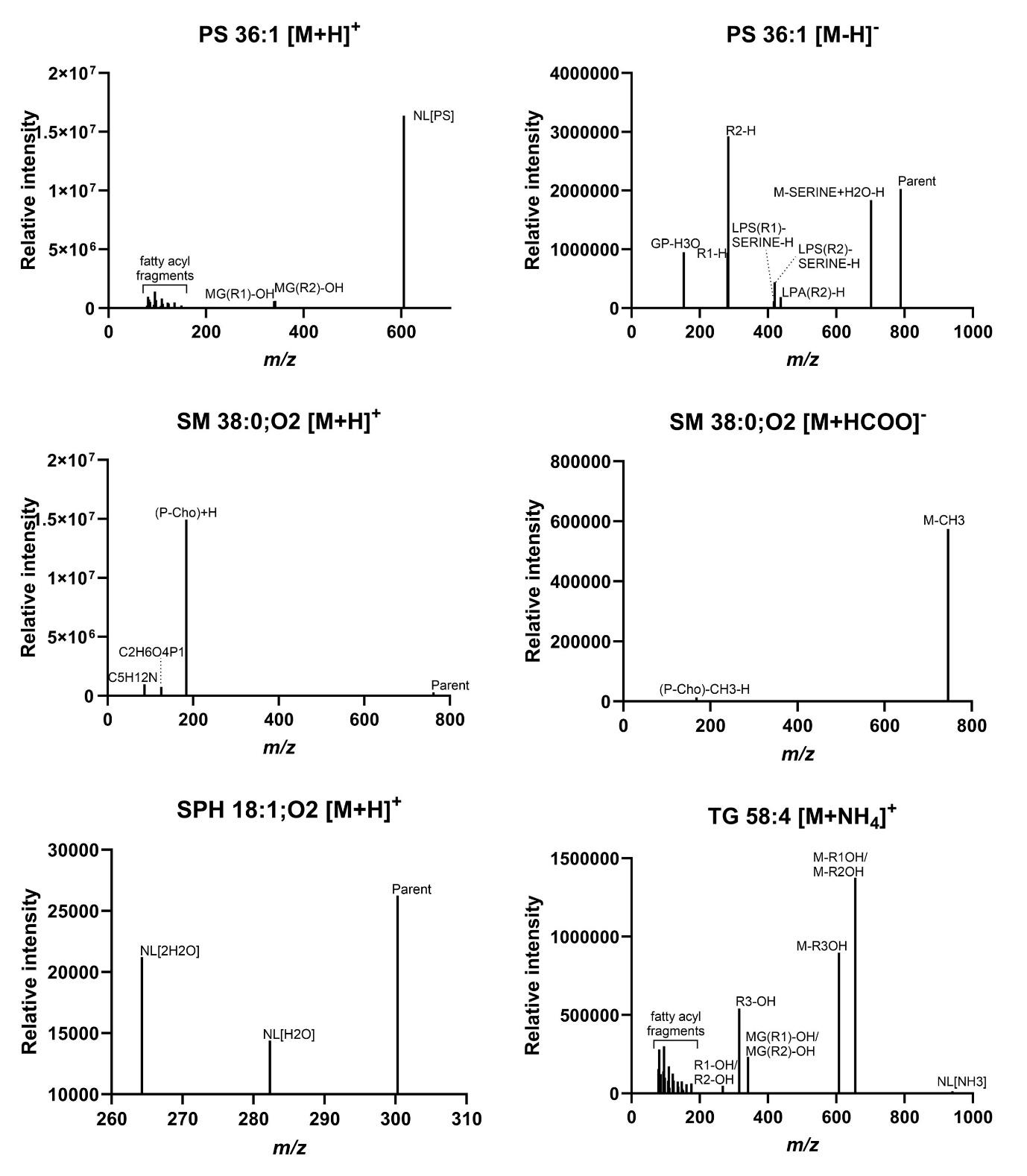


***Fig. S3*** *Representative MS/MS spectra for each lipid class measured in the cardiac microtissue lipid extracts. Plots show MS/MS fragments (measured m/z and their relative intensity) and their annotations as assigned by LipidSearch software, measured for the lipid species (lipid name and ion form) given in the plot header.*

# References

Archer CR, Sargeant R, Basak J, Pilling J, Barnes JR, Pointon A. (2018) Characterization and Validation of a Human 3D Cardiac Microtissue for the Assessment of Changes in Cardiac Pathology. Sci Rep. 8(1):10160.

Bowen, T.J., Hall, A.R., Lloyd, G.R., Weber, R.J.M., Wilson, A., Pointon, A. and Viant, M.R. (2021) An Extensive Metabolomics Workflow to Discover Cardiotoxin-Induced Molecular Perturbations in Microtissues. Metabolites 11, 644.

Chambers, M.C., Maclean, B., Burke, R., Amodei, D., Ruderman, D.L., Neumann, S., Gatto, L., Fischer, B., Pratt, B., Egertson, J., Hoff, K., Kessner, D., Tasman, N., Shulman, N., Frewen, B., Baker, T.A., Brusniak, M.Y., Paulse, C., Creasy, D., Flashner, L., Kani, K., Moulding, C., Seymour, S.L., Nuwaysir, L.M., Lefebvre, B., Kuhlmann, F., Roark, J., Rainer, P., Detlev, S., Hemenway, T., Huhmer, A., Langridge, J., Connolly, B., Chadick, T., Holly, K., Eckels, J., Deutsch, E.W., Moritz, R.L., Katz, J.E., Agus, D.B., MacCoss, M., Tabb, D.L. and Mallick, P. (2012) A cross-platform toolkit for mass spectrometry and proteomics. Nat Biotechnol 30, 918-20.

Smith, C.A., Want, E.J., O'Maille, G., Abagyan, R. and Siuzdak, G. (2006) XCMS: processing mass spectrometry data for metabolite profiling using nonlinear peak alignment, matching, and identification. Anal Chem 78, 779-87.

Sostare, E., Lawson, T.N., Saunders, L.R., Colbourne, J.K., Weber, R.J.M., Sobanski, T. and Viant, M.R. (2022) Knowledge-driven approaches to create the MTox700+ metabolite panel for predicting toxicity. *Toxicological Sciences*.

1. Signal-to-noise ratio (SNR): the height ratio of each chromatographic peak to the average baseline intensity [↑](#footnote-ref-1)
2. Intensity ratio (IR): ratio of the total number of points at the top (>50% peak intensity) to the total number of points at the bottom (<50% peak intensity) of each chromatographic peak. [↑](#footnote-ref-2)
3. Valid peak ratio (VPR): the fraction of peaks detected in the group that pass both the SNR and IR thresholds [↑](#footnote-ref-3)
